# Experiences of Loneliness Across the Lifespan: A Systematic Review and Thematic Synthesis of Qualitative Studies

**DOI:** 10.1080/17482631.2023.2223868

**Published:** 2023-06-16

**Authors:** Phoebe E. McKenna-Plumley, Rhiannon N. Turner, Keming Yang, Jenny M. Groarke

**Affiliations:** a Centre for Improving Health-Related Quality of Life, School of Psychology, Queen’s University Belfast, Belfast, UK; b Centre for Identity and Intergroup Relations, School of Psychology, Queen’s University Belfast, Belfast, UK; c Department of Sociology, Durham University, Durham, UK; d School of Psychology, University of Galway, Galway, Ireland

**Keywords:** Loneliness, Qualitative research, Systematic review, Life course perspective, Emotions, Thematic synthesis

## Abstract

**Purpose:**

Loneliness is a fundamentally subjective experience that is common at various life stages. Studies have qualitatively explored loneliness, but a comprehensive overview is lacking. This research therefore provides a fine-grained review of studies on loneliness experiences across the lifespan.

**Methods:**

A systematic review and thematic synthesis were performed on studies that qualitatively investigated experiences of loneliness in people of any age from non-clinical populations. Sensitivity analysis assessed the impact of lower-quality studies and specific age groups on the findings.

**Results:**

Twenty-nine studies of 1,321 participants aged between 7 and 103 were included. Fifteen descriptive themes and three overarching analytical themes were developed: (1) Loneliness is both psychological and contextual, (2) Loneliness centres on feelings of meaningful connection and painful disconnection, and (3) Loneliness can exist in a general, pervasive sense or can relate to specific other people or relationship types. Some features were particularly pertinent to children, younger adults, and older adults, respectively.

**Conclusions:**

Loneliness involves a primarily aversive psychological experience of perceived disconnection which is linked to physical, personal, and socio-political contexts and can be pervasive or relate to specific relationships or relationship types. An awareness of context, life stage, and personal experiences is essential to understand loneliness.

## Introduction

Loneliness is a common experience which is associated with various adverse physical and mental health outcomes (Cacioppo et al., 2010, Hawkley & Cacioppo, 2010, Victor & Yang, 2012). Despite an increasing body of research focusing on loneliness, there is relatively little work exploring its lived experience. Loneliness is generally defined as an unpleasant and distressing subjective phenomenon which arises when one’s desired level of social relations differs from their actual level in number or quality (Perlman & Peplau, 1981). However, the research lacks an overarching subjective perspective, which is a significant gap in the field given that loneliness is an inherently subjective experience. Unlike age, blood pressure, or other objective phenomena, loneliness can only be definitively evaluated by asking a person whether they feel lonely. Weiss (1987) argued that although existing definitions of loneliness may be helpful, they fail to reflect the actual nature of loneliness, because they define it in terms of potential causes rather than the actual experience of being lonely. Studies based on these definitions may therefore obscure the ways that loneliness is actually experienced, potentially failing to capture aspects and idiosyncrasies of the construct. Indeed, eminent researchers in the field have recently noted that there is not yet widespread consensus on definitions of loneliness (Fried et al., 2020). A robust understanding of the phenomenon is key to achieving suitable definitions and informing approaches to studying and ameliorating loneliness.

It is clear that objective aspects of a person’s social network such as social isolation are not sufficient to explain loneliness (Coyle & Dugan, 2012, Matthews et al., 2016). People can also experience meaningful social relationships which cannot be objectively evidenced, for example experiencing a relationship with God (Hawkley & Cacioppo, 2010). This underscores the need to better understand the subjective experience of loneliness. The use of qualitative methods is particularly appropriate to this end given that they can capture the idiosyncrasies of lived experiences.

Loneliness is present at various life stages and perhaps most commonly in younger and older adulthood (Victor & Yang, 2012). Qualter and colleagues (2015) suggest that sources of loneliness differ across the lifespan in line with changes in social priorities and influences. For example, relational proximity is prioritized more in childhood, intimacy in adolescence and young adulthood, romantic partnership in adulthood, and relational and physical losses are more relevant in older adulthood. Other factors such as how individualistic a culture is have been found to influence the relationship between age and loneliness (Barreto et al., 2021). While qualitative research has explored the experience of loneliness in adults at various life stages, a holistic investigation of these experiences across the lifespan is lacking. Rokach (1988) carried out the largest study of its type, analysing accounts of 526 adults’ loneliest experiences and creating a model with major elements of self-alienation, interpersonal isolation, distressed reactions, and agony. However, most respondents were aged between 19 and 45 years old and the findings may be usefully considered alongside research qualitatively examining loneliness in wider age groups (e.g., Ojembe & Ebe Kalu, 2018). While cohort studies are useful to examine change across the lifespan, synthesizing research from different age groups can also elucidate the experience of loneliness and how it may be stable or differ over the lifespan.

Existing systematic reviews have explored the conceptualizations of loneliness employed in qualitative research with adults (Mansfield et al., 2019), experiences of loneliness in young people with depression (Achterbergh et al., 2020), and experiences and ways of dealing with loneliness in older adults published in healthcare journals (Kitzmüller et al., 2018). In contrast, the present systematic review takes a data-driven approach that focuses on non-clinical populations of all ages to synthesize participants’ lived experiences of loneliness. This knowledge is imperative to deepen current understandings of loneliness and inform approaches to describing, explaining, and potentially ameliorating it. This review therefore aims to offer a holistic view of the experience of loneliness across the lifespan. There is one research question: How do people describe their experiences of loneliness?

## Method

The full review protocol has been published (McKenna-Plumley et al., 2020) and registered on PROSPERO (registration number: CRD42020178105). Ethical approval was not required as primary data were not collected. The review is reported in accordance with the Enhancing Transparency in Reporting the Synthesis of Qualitative Research (ENTREQ) statement (Tong et al., 2012). The inclusion criteria were as follows:

1. Studies published in English
2. Studies with a qualitative component (reported separately if quantitative methods are also used)
3. Studies of individuals of any age who describe experiences of loneliness, not including specific clinical populations, settings, or samples closely adjacent to clinical populations (see “Amendments to protocol” for details)
4. Studies which report the subjectively perceived experience of loneliness
5. Studies in which the primary focus or one of the primary focuses is experiences of loneliness. Specifically, papers were deemed to have a sufficient focus if studying loneliness experiences was a key aspect of the work rather than simply part of the output. Studies were included if authors stated a relevant aim, objective, or research question related to investigating experiences of loneliness or if loneliness experiences were clearly explored and described (e.g., relevant questions were present in an interview guide). At the title and abstract screening stage, at least one relevant sentence or information that indicated likely relevance had to be present for inclusion.
6. Studies published in peer-reviewed journals

### Amendments to protocol

Through familiarization with the available evidence, several refinements were necessary to complete a thorough and well-conceptualized review. The amendments are as follows:

1. The inclusion criterion regarding clinical populations was further specified such that clinical settings and closely clinically adjacent populations were also excluded. This was refined after noting the wide range of populations in the literature, which included specific groups adjacent to clinical populations experiencing the same types of specific circumstances as those populations. Clinical populations were defined as those with a specific clinical issue included in the ICD−11 (World Health Organization, 2021). Clinical settings included, for example, hospitals and nursing homes. Samples closely adjacent to clinical populations included, for example, carers, people experiencing bereavement, intervention participants, and individuals experiencing abuse and secondary trauma.
2. Studies in which a proportion of participants were part of a specific clinical or clinically adjacent population or setting were included if results concerning these individuals were presented separately.
3. Grey literature and books were excluded following title and abstract screening as the volume of peer-reviewed articles was sufficient to enable a substantial but thorough qualitative synthesis.
4. The plan to use the GRADE-CERQual approach to assess confidence in findings was removed as the approach is intended primarily for findings to be used in decision-making processes (Lewin et al., 2018) and is not as applicable to the many findings of the present review which stem closely from the analysis and are not highly context specific.

### Information sources and search strategy

Peer-reviewed literature was gathered between the 10^th^ and 12^th^ of March, 2021 from searches of PsycINFO, MEDLINE, Scopus, Child Development & Adolescent Studies, Sociological Abstracts, International Bibliography of the Social Sciences (IBSS), CINAHL, and the Education Resource Information Center (ERIC). Literature from database inception until the search date was considered. Additional searches for grey and difficult-to-locate literature were completed using Google Scholar, opengrey.eu, ProQuest Dissertations and Theses, and websites of specific loneliness organizations, although grey literature was later removed (see “Amendments to Protocol”). In collaboration with information specialists, the search strategy was developed and search terms were translated to be appropriate for each database. The search strategy was peer reviewed by an information specialist using the Peer Review of Electronic Search Strategies (PRESS) Checklist (McGowan et al., 2016). The completed PRESS checklist is provided in Additional File 1 and the search strategy for MEDLINE is provided in Additional File 2. To find additional studies, reference lists of included studies were checked, as were the reference lists of similar systematic reviews (Kitzmüller et al., 2018, Mansfield et al., 2019, Shorey & Chan, 2021), and authors of included studies were asked to refer relevant studies.

### Study selection

The database search, hand-searching, and initial screening for duplicates were completed by PMP. Title and abstract screening to remove articles which were irrelevant or inapplicable to the inclusion criteria was carried out by PMP with JG independently screening 50.92% of all titles and abstracts in Rayyan (Ouzzani et al., 2016). Full-text versions of the remaining articles were screened by PMP with JG independently reviewing 50% of the full-texts. In cases of disagreement, the two reviewers discussed the study to reach a decision about inclusion or exclusion. The reason for the exclusion at the full-text stage was recorded.

### Data extraction and quality appraisal

Information about study characteristics was extracted by PMP and fully checked by JG using a purpose-designed and piloted Microsoft Excel form. Lead authors were contacted to specify missing information. In line with guidance from Thomas and Harden (2008), all text labelled as “results” or “findings” was extracted and entered into NVivo software for analysis.

Quality of the included articles was assessed by PMP using the Joanna Briggs Institute (JBI) Critical Appraisal Checklist for Qualitative Research (Lockwood et al., 2015). Lower-quality studies were included in the review but taken into account during sensitivity analysis.

### Analysis

Thematic synthesis was used to synthesize the results of the review. In thematic synthesis, descriptive themes which remain close to the primary study findings are developed. Following this, analytical theme development can be undertaken to go beyond the interpretations of primary studies and develop higher-order explanations (Thomas & Harden, 2008).

**Figure 1.**
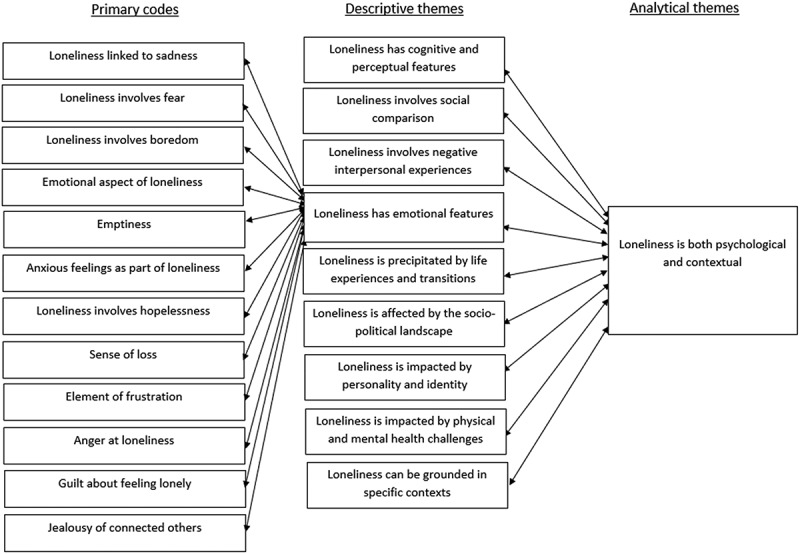
Example of reciprocal development from codes to descriptive themes to analytical themes within thematic synthesis.

The data were coded line-by-line by PMP with codes created inductively to represent the data. Most codes represented semantic features of the data; some captured more latent aspects. Studies were coded in alphabetical order by first author’s surname and then a second time working backwards from the alphabetical midpoint to check, add to, and refine codes. This ensured a relatively equal focus of coding across studies. Codes were systematically and inductively organized into descriptive themes which remained relatively close to the data. Following this, the descriptive themes were further interpreted to develop analytical themes representing overarching answers to the research question, going beyond the findings of the original studies. The process was recursive, with codes and candidate themes refined and revised as the analysis developed. Final themes were established through discussion between PMP and JG. Illustrative quotes from original study participants are provided in italics to remain grounded in the data. The relationship between codes, descriptive themes, and analytical themes is visualized in Figure 1, showing an example of the descriptive themes which primarily contributed to the development of one analytical theme and the codes underpinning one of these descriptive themes.

Following synthesis, sensitivity analyses were carried out to assess if and how (i) lower-quality studies and (ii) studies of specific age groups (children, young adults, middle-aged adults, and older adults) impacted the synthesis. This involved removing studies with specific characteristics from the database to assess whether the synthesis is altered by their removal, in terms of any themes being lost entirely or becoming less rich or thick (Carroll & Booth, 2015). We respectively removed lower-quality studies and studies of specific age groups to assess the impact of these characteristics on the findings.

## Results

**Figure 2.**
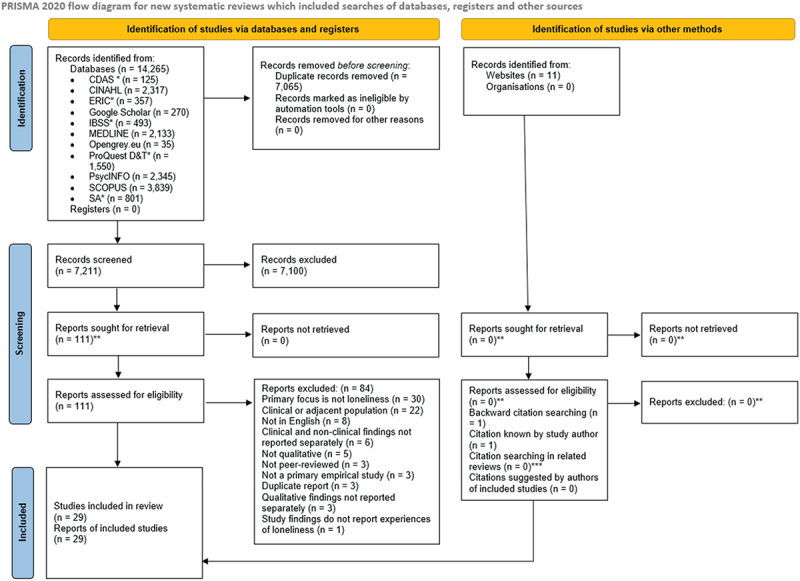
PRISMA 2020 flow diagram.*Acronyms: CDAS = Child Development & Adolescent Studies; ERIC = Education Resource Information Center; IBSS = International Bibliography of the Social Sciences; ProQuest D&T = ProQuest Dissertations & Theses; SA = Sociological Abstracts **Prior to full-text screening, the decision was made to exclude studies that were not published in peer-reviewed journals (i.e. grey literature, books, book chapters). ***Reviews searched: Kitzmüller et al., 2018; Mansfield et al., 2019; Shorey & Chan, 2021.

Of the 14,276 citations retrieved by the search, 7,065 were duplicates, leaving 7,211 for title and abstract screening. After title and abstract screening, the decision to limit the review to articles published in peer-reviewed journals was made. Following these stages, 111 articles remained for full-text screening and 29 studies were included in the final review. Where multiple reports of the same study were found, the most comprehensive was included in the review. A flowchart of the number of studies included and excluded at each stage of this process is shown in the PRISMA 2020 flow diagram (Page et al., 2021) in Figure 2.

The 29 distinctive studies included a total of 1,321 participants aged from 7 to 103 years old. A breakdown of age categories was performed for later sensitivity analysis. The categorization was based on theoretical considerations regarding major developmental stages, namely Erikson’s (1950) psychosocial development theory and Arnett’s (2015) theory of emerging adulthood, and alignment with related research (Luhmann & Hawkley, 2016, Rokach, 2000, Victor & Yang, 2012, Yang & Victor, 2011). Five studies included only children (0–15 years; Berguno et al., 2004, Karnick, 2008, Kirova-Petrova, 2000, Kristensen, 1995, Martin et al., 2014), two studies included only young adults (16–29 years; Firmin et al., 2014, Rönkä et al., 2018), and eleven studies included only older adults (60 years and older; Graneheim & Lundman, 2010, Heravi-Karimooi et al., 2010, Kwegyir Tsiboe, 2021, McInnis & White, 2001, Morgan & Burholt, 2020, Ojembe & Ebe Kalu, 2018, Park et al., 2019, Smith, 2012, Sullivan et al., 2016, Tiilikainen & Seppänen, 2017, Wright-St Clair & Nayar, 2020). No studies included only middle-aged adults (30–59 years). Eleven studies included multiple age groups, but only one (Dahlberg, 2007) included participants from all age groups.

Studies were published between 1988 and 2021 and carried out in England (*N* = 4), Sweden (*N* = 3), the USA (*N* = 3), Finland (*N* = 3), Canada (*N* = 2), Australia (*N* = 2), New Zealand (*N* = 2), Italy (*N* = 1), Ghana (*N* = 1), Nigeria (*N* = 1), Wales (*N* = 1), Iran (*N* = 1), the UK (*N* = 1), and North America (*N* = 1). Three studies did not specify a location.

Several studies included populations that were specified by characteristics beyond age, including migrants, first-time mothers, homeless adolescents, university students in long-distance relationships, and older people living alone. Full study characteristics are presented in Table I.

**Table I.** Characteristics of included studies (*N* = 29).

#	Study	Location	Method	Analysis	Population	Participant N	Participant Age (Years)	Were participants directly asked about loneliness?	JBI Quality Rating
1	Barke (2017)	Greater Bedminister area of Bristol, England	Interviews	Thematic analysis	Local people aged 50+	14	52–88	Unspecified	8
2	Berguno et al. (2004)	West London, Central London and Brighton areas, England	Semi-structured interviews	Empirical-phenomenological method	Primary school children	42	8–10	Yes	7
3	Cela and Fokkema (2017)	Marche region, Italy	Semi-structured interviews	Thematic analysis	Older Albanian and Moroccan migrants in Italy	34	51–84	Yes	4
4	Dahlberg (2007)	Sweden	Interviews	Dynamic analysis to describe a structure of meanings that characterize the phenomenon	Non-specific	26	12–82	Yes	8
5	Davidson (1995)	New Brunswick, Canada	Semi-structured interviews and validation focus group	Grounded theory	White, middle-class, adolescent women	10	15–18	Unspecified	9
6	Firmin et al. (2014)	Midwestern USA	Semi-structured interviews	Thematic analysis	Female sophomore college students involved in heterosexual distance relationships	16	18–21	Yes	8
7	Graneheim and Lundman (2010)	Northern Sweden	Thematic interviews	Qualitative content analysis	People over the age of 85 who live alone in a specific area in Northern Sweden	30	85–103	Yes	3
8	Hemberg et al. (2021)	Southern and west-coast parts of Finland	Remote semi-structured interviews	Content analysis	Swedish-speaking Finns between 17 and 30 years old	15	17–30	Yes	10
9	Heravi-Karimooi et al. (2010)	Tehran, Iran	In-depth semi-structured interviews	Elements of descriptive and interpretive phenomenology	Adults over the age of 65	13	68–87	Yes	9
10	Herz and Lalander (2017)	Sweden	Repeated interviews and observations	Process of theme identification following from symbolic interactionism and ethnography	Young people who have arrived in Sweden without their parents or legal guardians	23	15–25	Yes	10
11	Karnick (2008)	Unspecified	Dialogical engagement with participants involving conversation and drawing	Extraction-synthesis process of constructing a story of essential ideas and structure of experiences (heuristic interpretation)	Children	10	7–10	Unspecified	10
12	Kirova-Petrova (2000)	Canada	Board game-facilitated conversation	Phenomenological approach to theme identification	Linguistically diverse schoolchildren	10	8–10	Yes	9
13	Kristensen (1995)	Midwestern USA	Unstructured interviews and drawing	Phenomenological method described by Spiegelberg (1982)	Children from middle-class White families	14	8–10	Yes	7
14	Kwegyir Tsiboe (2021)	Emmena—Ashanti region, Ghana	Semi-structured interviews	Thematic analysis	Older people in rural Ghana	10	60–80	Yes	10
15	Lee et al. (2019)	Bath, England	Semi-structured interviews	Interpretative phenomenological analysis	First-time mothers	7	25–44 (age range)	Yes	9
16	Martin et al. (2014)	Western Australia, Australia	Semi-structured interviews	Conventional content analysis with constant comparative coding	Early- to mid-aged adolescents	33	10–15	Yes	3
17	McInnis and White (2001)	Unspecified	Interviews	Giorgi’s (1985) phenomenological method	Older adults	20	71–85	Yes	9
18	McKenna-Plumley et al. (2021)	UK	Semi-structured remote interviews	Reflexive thematic analysis	Adults who had felt lonely during COVID − 19 lockdown	8	21–67	Yes	10
19	Morgan & Burholt, (2020)	Wales	Narrative interviews	Interpretative phenomenological analysis	Older people who had self-described as sometimes or always lonely	11	67–84	Yes	9
20	Ojembe and Ebe Kalu (2018)	Port Harcourt Metropolis, Nigeria	Semi-structured interviews	Inductive thematic analysis	Older adults in Nigeria	12	62–88	Yes	9
21	Park et al. (2019)	Auckland, New Zealand	Interviews and focus groups	Thematic analysis approach	Older Asian migrants in New Zealand and Chinese professionals or service providers	25	65+	Yes	8
22	Rew (2000)	Central Texas, USA	Focus groups and semi-structured interviews	Manifest and latent content analysis	Homeless adolescents	42	15–23	Yes	3
23	Rokach (1988)	North America	Written accounts of loneliness, short meetings, and informal discussions	Content analysis	Non-specific	526	16–84	Yes	8
24	Rönkä et al. (2018)	Finland	Semi-structured interviews	Qualitative content analysis	Young adults born in Northern Finland	35	27–28	Yes	9
25	Sawir et al., (2008)	Australia	Interviews	Unspecified	International students in Australia	200	Unspecified	Yes	2
26	Smith (2012)	Unspecified	Multiple structured interviews	Interpretative phenomenological analysis with thematic analysis	Community-dwelling older adults	12	74–98	Yes	9
27	Sullivan et al. (2016)	South England	In-depth interviews	Thematic analysis	Older people who had self-described as lonely or sometimes lonely	37	65–87	Yes	5
28	Tiilikainen and Seppänen (2017)	Päijät-Häme, southern Finland	In-depth interviews	Analysis based on Layder’s (1998) model of adaptive theory	Older people	10	70–84	Yes	9
29	Wright-St Clair and Nayar (2020)	Auckland, New Zealand	Focus groups and interviews	Secondary analysis following Gadamer’s (2004) hermaneutic conditions	Chinese, Indian, and Korean late-life immigrants in Auckland, New Zealand	76	60–83	No	10

*Note: The review also set out to consider the reasons that study authors offered for the relative shortage of qualitative work. No specific reasons were given.

### Findings

**Figure 3.**
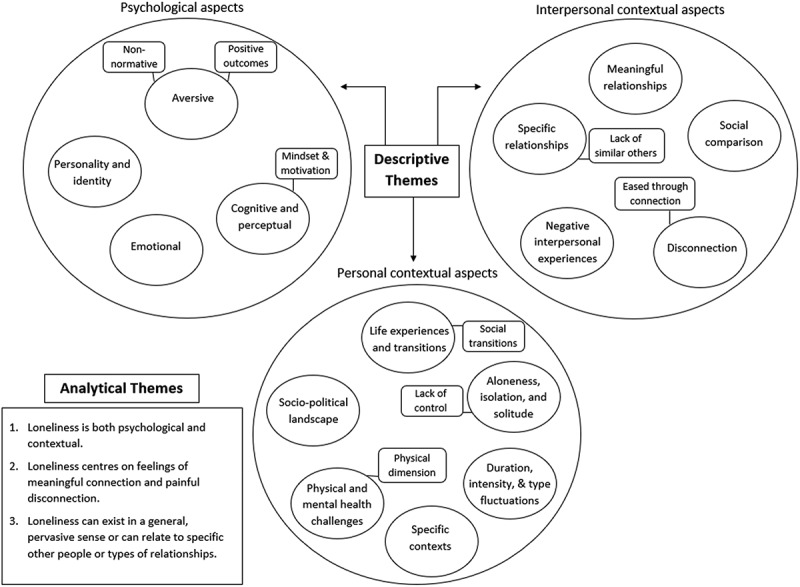
Analytical themes and descriptive themes organized into psychological, interpersonal-contextual, and personal-contextual domains.

Fifteen descriptive themes which synthesize the findings of the primary studies were developed. The descriptive themes are organized into three overarching domains; specifically, in describing experiences of loneliness, people tended to discuss its (i) psychological aspects, (ii) interpersonal contextual aspects, and (iii) personal contextual aspects. The fifteen descriptive themes were then further interpreted to create three analytical themes which describe higher-order explanations in response to the research question. The descriptive and analytical themes are displayed in Figure 3. The studies contributing data to each descriptive theme are displayed in Table II.

**Table II.** Studies contributing to descriptive themes.

	Psychological Aspects				Interpersonal Contextual Aspects					Personal Contextual Aspects					
Study	Loneliness is an aversive experience	Loneliness has emotional features	Loneliness has cognitive and perceptual features	Loneliness is impacted by personality and identity	Loneliness can relate to specific relationships (or their absence)	Loneliness relates to a lack of close, meaningful relationships—not superficial connections	Loneliness involves feelings of disconnection	Loneliness involves negative interpersonal experiences	Loneliness involves social comparison	Loneliness is connected to, but separate from, aloneness, isolation, and solitude	Loneliness is precipitated by life experiences and transitions	Loneliness fluctuates in duration, intensity, and type	Loneliness can be grounded in specific contexts	Loneliness is impacted by physical and mental health challenges	Loneliness is affected by the socio-political landscape
Barke (2017)	*				*	*	*	*	*	*	*	*	*	*	*
Berguno et al. (2004)		*				*	*	*		*	*	*	*		
Cela and Fokkema (2017)	*	*	*	*	*	*	*	*	*	*	*	*	*	*	*
Dahlberg (2007)	*	*	*	*	*	*	*	*	*	*	*	*	*	*	*
Davidson (1995)	*	*	*		*	*	*	*	*	*	*	*	*		
Firmin et al. (2014)	*	*	*		*	*	*	*	*	*	*	*	*	*	
Graneheim and Lundman (2010)	*	*	*		*	*	*	*		*	*	*		*	
Hemberg et al. (2021)	*	*	*		*	*	*	*	*	*	*	*	*	*	*
Heravi-Karimooi et al. (2010)	*	*			*	*	*	*	*	*	*	*		*	*
Herz and Lalander (2017)			*	*	*	*	*	*		*	*	*	*		*
Karnick (2008)	*	*	*			*	*	*	*	*	*	*	*		
Kirova-Petrova (2000)	*	*	*		*	*	*	*	*	*			*	*	
Kristensen (1995)	*	*			*	*	*	*		*	*	*	*	*	
Kwegyir Tsiboe (2021)	*		*	*	*	*	*	*		*	*	*	*	*	*
Lee et al. (2019)		*	*		*	*	*	*	*	*	*		*	*	*
Martin et al. (2014)	*	*			*	*	*	*		*	*	*			
McInnis and White (2001)	*	*	*	*	*	*	*	*	*	*	*	*	*	*	*
McKenna-Plumley et al. (2021)	*	*	*	*	*		*			*	*	*		*	*
Morgan & Burholt, (2020)	*		*	*	*		*		*	*	*	*	*	*	*
Ojembe and Ebe Kalu (2018)	*	*		*	*	*	*			*	*	*		*	*
Park et al. (2019)	*	*	*	*	*			*		*	*		*	*	*
Rew (2000)	*	*		*	*	*	*	*		*	*	*	*	*	
Rokach (1988)	*	*	*		*	*	*	*	*	*	*		*	*	*
Rönkä et al. (2018)	*	*	*	*	*	*	*	*	*	*	*	*	*	*	*
Sawir et al., (2008)	*	*	*	*	*	*	*	*	*	*	*	*	*	*	*
Smith (2012)	*	*	*	*	*	*	*	*		*	*	*	*	*	*
Sullivan et al. (2016)	*		*	*	*	*	*	*	*	*	*	*	*	*	*
Tiilikainen and Seppänen (2017)	*	*	*		*	*	*	*	*	*	*	*	*	*	*
Wright-St Clair and Nayar (2020)	*			*	*	*	*	*		*	*		*	*	*

#### Descriptive themes

The 15 descriptive themes representing a synthesis of the primary study findings are described below under overarching domains of psychological, interpersonal-contextual, and personal-contextual aspects of the loneliness experience. Several themes include related subthemes, represented with subheadings to express distinct but cohesive parts of the wider theme.

### Psychological aspects of the experience

The following four descriptive themes represent common psychological aspects of the loneliness experience as described by people who have experienced the feeling.

## Loneliness is an aversive experience

*Loneliness is like a nasty disease that eats us up little by little, day by day.* (Cela & Fokkema, 2017)

Loneliness was generally described as a negative experience by participants at ages across the lifespan (Barke, 2017, Cela & Fokkema, 2017, Dahlberg, 2007, Davidson, 1995, Firmin et al., 2014, Graneheim & Lundman, 2010, Hemberg et al., 2021, Heravi-Karimooi et al., 2010, Kristensen, 1995, Martin et al., 2014, McInnis & White, 2001, Ojembe & Ebe Kalu, 2018, Park et al., 2019, Rokach, 1988, Rönkä et al., 2018, Smith, 2012, Sullivan et al., 2016). It was commonly depicted as painful (Dahlberg, 2007, Heravi-Karimooi et al., 2010, Kirova-Petrova, 2000, Kristensen, 1995, Kwegyir Tsiboe, 2021, Martin et al., 2014, Rokach, 2000, Rönkä et al., 2018) and could even provoke suicidal thoughts (Graneheim & Lundman, 2010, Hemberg et al., 2021, Kwegyir Tsiboe, 2021, Ojembe & Ebe Kalu, 2018, Rönkä et al., 2018, Tiilikainen & Seppänen, 2017). There were clear indications that people would attempt to avoid loneliness through strategies such as blocking it out and keeping busy (Cela & Fokkema, 2017, Dahlberg, 2007, Davidson, 1995, Firmin et al., 2014, Hemberg et al., 2021, Karnick, 2008, Kristensen, 1995, Martin et al., 2014, McInnis & White, 2001, McKenna-Plumley et al., 2021, Park et al., 2019, Rew, 2000, Rokach, 1988, Rönkä et al., 2018, Sawir et al., 2008, Smith, 2012, Sullivan et al., 2016, Wright-St Clair & Nayar, 2020). There was an additional drive to stop loneliness from negatively impacting upon others, making people hesitant to discuss their loneliness with others (Cela & Fokkema, 2017, Davidson, 1995, Firmin et al., 2014, Hemberg et al., 2021, Karnick, 2008, McInnis & White, 2001, McKenna-Plumley et al., 2021, Sawir et al., 2008, Sullivan et al., 2016). Its aversive nature was reflected in fears about future loneliness (Barke, 2017, Cela & Fokkema, 2017, McInnis & White, 2001).

### Loneliness feels non-normative

Loneliness was experienced as shameful, stigmatized, and a sign that something is wrong with you (Cela & Fokkema, 2017, Dahlberg, 2007, Davidson, 1995, Hemberg et al., 2021, Kirova-Petrova, 2000, McInnis & White, 2001, Rew, 2000, Rokach, 1988, Sullivan et al., 2016). This was linked to the sense that being isolated was not normal (Dahlberg, 2007, Hemberg et al., 2021, Martin et al., 2014, McKenna-Plumley et al., 2021, Rokach, 1988, Rönkä et al., 2018, Wright-St Clair & Nayar, 2020) and that humans are inherently social (Dahlberg, 2007, Hemberg et al., 2021, Wright-St Clair & Nayar, 2020).

### The potential for positive outcomes

Some studies referred to positive forms, interpretations, or outcomes (Dahlberg, 2007, Karnick, 2008). A “voluntary loneliness” (Dahlberg, 2007) mentioned in one study (which may also be referred to as solitude) could include pleasant, restful feelings. However, this aspect did not usually refer to positive experiences of feeling lonely. Instead, it generally involved a positive reframing of loneliness (for example, as an opportunity for self-improvement) or positive outcomes such as nurturing creativity (Dahlberg, 2007; Firmin et al., 2014; Graneheim & Lundman, 2010; Hemberg et al., 2021; Karnick, 2008; Morgan & Burholt, 2020; Rönkä et al., 2018; Sawir et al., 2008; Sullivan et al., 2016).

## Loneliness has emotional features

*I would say it is sadness; that is the best word to describe it.* (Rönkä et al., 2018)

The emotions that came alongside loneliness were a key aspect of the experience. Sadness was most commonly described as part of the loneliness experience (Cela & Fokkema, 2017, Dahlberg, 2007, Davidson, 1995, Heravi-Karimooi et al., 2010, Karnick, 2008, Kirova-Petrova, 2000, Martin et al., 2014, McInnis & White, 2001, McKenna-Plumley et al., 2021, Park et al., 2019, Rokach, 1988, Rönkä et al., 2018, Sawir et al., 2008, Smith, 2012, Tiilikainen & Seppänen, 2017). Additionally, loneliness included boredom, as well as fear, emptiness, anxious feelings, hopelessness, and a feeling of loss (Cela & Fokkema, 2017, Dahlberg, 2007, Firmin et al., 2014, Graneheim & Lundman, 2010, Hemberg et al., 2021, Heravi-Karimooi et al., 2010, Karnick, 2008, Kirova-Petrova, 2000, Kristensen, 1995, McInnis & White, 2001, McKenna-Plumley et al., 2021, Ojembe & Ebe Kalu, 2018, Park et al., 2019, Rew, 2000, Rokach, 1988, Rönkä et al., 2018, Smith, 2012, Tiilikainen & Seppänen, 2017). Anger and frustration regarding the experience of loneliness were sometimes reported (Hemberg et al., 2021, Karnick, 2008, Kristensen, 1995, Lee et al., 2019, McKenna-Plumley et al., 2021, Rokach, 1988). In some cases, guilt and jealousy were also expressed (Firmin et al., 2014, McKenna-Plumley et al., 2021).

## Loneliness has cognitive and perceptual features

*[T]he shame for being lonely. … it’s in some way connected with being weak and with being something wrong with you so that … you should blame yourself for being alone.* (Hemberg et al., 2021)

Loneliness involved a range of cognitive and perceptual features. It was related to thought processes including self-blame, where loneliness was attributed to their characteristics or behaviours (Davidson, 1995, Hemberg et al., 2021, McInnis & White, 2001, Rönkä et al., 2018, Sawir et al., 2008), and existential concerns about life and belonging (Cela & Fokkema, 2017, Dahlberg, 2007, Davidson, 1995, Rokach, 1988, Sawir et al., 2008, Tiilikainen & Seppänen, 2017).

Self-perception could also be impacted by loneliness, making people feel inferior (Herz & Lalander, 2017, Karnick, 2008, Kirova-Petrova, 2000, McInnis & White, 2001, Morgan & Burholt, 2020, Rokach, 1988). Loneliness could damage self-esteem, which was also a causal factor for loneliness, and bring about self-contempt (Dahlberg, 2007, Davidson, 1995, Graneheim & Lundman, 2010, Hemberg et al., 2021, Karnick, 2008, Kirova-Petrova, 2000, Lee et al., 2019, Morgan & Burholt, 2020, Park et al., 2019, Rokach, 1988, Rönkä et al., 2018, Sawir et al., 2008, Smith, 2012). Perceptions of incompleteness and lack of meaning in life were also salient (Graneheim & Lundman, 2010, Hemberg et al., 2021, Smith, 2012, Sullivan et al., 2016, Tiilikainen & Seppänen, 2017).

Loneliness experiences could additionally involve altered perceptions of the physical world including feelings of coldness (Hemberg et al., 2021, Rokach, 1988, Sullivan et al., 2016) and altered time perception where time could pass too quickly, too slowly, or stop (Dahlberg, 2007, Hemberg et al., 2021, Kirova-Petrova, 2000, Rokach, 1988).

### Mindset and motivation impact loneliness

Loneliness could cause a negative mindset (Firmin et al., 2014, Hemberg et al., 2021, Karnick, 2008), but people suggested that a positive mindset could counteract loneliness (Davidson, 1995, Firmin et al., 2014, Graneheim & Lundman, 2010, Karnick, 2008, Kwegyir Tsiboe, 2021, McInnis & White, 2001, Morgan & Burholt, 2020, Park et al., 2019, Tiilikainen & Seppänen, 2017).

*It’s a lot of positive thinking. I couldn’t get through without talking to myself.* (Davidson, 1995)

Cognitive approaches including adjusting one’s thinking and internalizing a positive outlook could ease loneliness. Lonely individuals, particularly older ones, suggested that coping with loneliness was one’s own responsibility (Barke, 2017, Davidson, 1995, McInnis & White, 2001, Morgan & Burholt, 2020, Rönkä et al., 2018, Sullivan et al., 2016, Wright-St Clair & Nayar, 2020). However, loneliness could create a passive or negative attitude towards socializing, which could reinforce loneliness by reducing the inclination or ability to connect or otherwise manage the feeling (Berguno et al., 2004, Cela & Fokkema, 2017, Dahlberg, 2007, Davidson, 1995, Kirova-Petrova, 2000, Kristensen, 1995, Lee et al., 2019, Martin et al., 2014, McInnis & White, 2001, Morgan & Burholt, 2020, Rokach, 1988, Smith, 2012).

## Loneliness is impacted by personality and identity

*“I am a lonely person.”* (Sullivan et al., 2016)

Personality and identity could affect one’s propensity for loneliness. Personality traits like introversion and timidness were given as reasons for loneliness and isolation (Dahlberg, 2007, McKenna-Plumley et al., 2021, Rönkä et al., 2018). For some, loneliness was considered part of their character (Rew, 2000, Rönkä et al., 2018, Sawir et al., 2008, Sullivan et al., 2016). An isolated identity was also relevant, for example for young people labelled as “unaccompanied” and older adults who were perceived as weak (Dahlberg, 2007, Herz & Lalander, 2017, Kwegyir Tsiboe, 2021, Ojembe & Ebe Kalu, 2018). Two studies of lonely older adults indicated that when long-lived identities (such as partner, parent, or worker) became unavailable, loneliness could occur (Morgan & Burholt, 2020, Smith, 2012). Religion also served an important role for some by providing an identity, community, and purpose (Cela & Fokkema, 2017, Kwegyir Tsiboe, 2021, McInnis & White, 2001, Morgan & Burholt, 2020, Ojembe & Ebe Kalu, 2018, Park et al., 2019, Rönkä et al., 2018, Sawir et al., 2008, Wright-St Clair & Nayar, 2020).

### Interpersonal contextual aspects of the experience

The following five descriptive themes represent aspects of the loneliness experience related to the interpersonal context.

## Loneliness can relate to specific relationships (or their absence)

*Friendship is so very important; good, true friendships are very important. That is the greatest antidote to loneliness.* (Barke, 2017)

While loneliness was sometimes described in a general, pervasive, or non-specific sense, many descriptions invoked specific types of relationships. Loneliness was often related to not having friends, whether or not other relationships (such as family or romantic partners) were available (Barke, 2017, Berguno et al., 2004, Cela & Fokkema, 2017, Davidson, 1995, Firmin et al., 2014, Hemberg et al., 2021, Herz & Lalander, 2017, Kirova-Petrova, 2000, Martin et al., 2014, Rew, 2000, Rokach, 1988, Rönkä et al., 2018, Sawir et al., 2008, Tiilikainen & Seppänen, 2017, Wright-St Clair & Nayar, 2020). Friends were people to have fun with, confide in, and receive emotional support from. A lack of satisfying family relationships was also implicated, primarily by older adults with respect to their adult children (Barke, 2017, Cela & Fokkema, 2017, Heravi-Karimooi et al., 2010, Herz & Lalander, 2017, Kwegyir Tsiboe, 2021, Lee et al., 2019, McInnis & White, 2001, Morgan & Burholt, 2020, Ojembe & Ebe Kalu, 2018, Park et al., 2019, Rönkä et al., 2018, Smith, 2012, Tiilikainen & Seppänen, 2017, Wright-St Clair & Nayar, 2020). Family provided instrumental care and social support which were missed when absent.

*I can’t remember the last time, my son or even his friends visited me, he lives 5 minutes away by car. They are only interested in sending me money, which is not really what I need.* (Ojembe & Ebe Kalu, 2018)

Romantic partners were also relevant (Dahlberg, 2007, Heravi-Karimooi et al., 2010, Rew, 2000, Rokach, 1988, Rönkä et al., 2018, Tiilikainen & Seppänen, 2017). Some studies mentioned that being single could feel abnormal in a heteronormative society, as emphasis is placed upon romantic partnership with a member of the “opposite” sex. Lonely individuals who had lost partners reported the desire for a new partner for companionship and emotional intimacy (though not universally) (Heravi-Karimooi et al., 2010, Tiilikainen & Seppänen, 2017). Lacking a social network could also be lonely (Barke, 2017, Cela & Fokkema, 2017, Herz & Lalander, 2017, Kirova-Petrova, 2000, Lee et al., 2019, Ojembe & Ebe Kalu, 2018, Park et al., 2019, Rönkä et al., 2018, Sawir et al., 2008, Wright-St Clair & Nayar, 2020). Loneliness could be caused by missing specific people, such as deceased spouses or friends left behind after migration (Barke, 2017, Cela & Fokkema, 2017, Firmin et al., 2014, Graneheim & Lundman, 2010, Herz & Lalander, 2017, Kirova-Petrova, 2000, Kristensen, 1995, Kwegyir Tsiboe, 2021, Lee et al., 2019, McKenna-Plumley et al., 2021, Rokach, 1988, Smith, 2012, Tiilikainen & Seppänen, 2017).

*I wanted my mum and I wanted my family around me a lot more so I felt lonely for them.* (Lee et al., 2019)

Animal connections were also mentioned in relation to easing loneliness (Dahlberg, 2007, Davidson, 1995, Kwegyir Tsiboe, 2021, Rew, 2000, Smith, 2012, Sullivan et al., 2016).

Differences between family, friend, and romantic relationships were mentioned (Cela & Fokkema, 2017, Kwegyir Tsiboe, 2021, Lee et al., 2019, Rönkä et al., 2018, Sullivan et al., 2016, Tiilikainen & Seppänen, 2017, Wright-St Clair & Nayar, 2020). Lonely older adults could not talk to their children about loneliness, whereas friends could understand and advise (Cela & Fokkema, 2017, McInnis & White, 2001). For new mothers, social hierarchies shifted and family connections became most valuable (Lee et al., 2019). One study noted differences based on differing religious norms around the acceptability of remarrying and having multiple spouses (Kwegyir Tsiboe, 2021).

There was also overlap. Some individuals described missing multiple types of relationships and for some the same people played multiple social roles, such as boyfriend and best friend. However, for many, loneliness stemmed from missing specific people or lacking specific types of relationships.

### Lack of close relationships with similar others

Loneliness could also arise due to a lack of relationships with people who shared certain characteristics. People in various age groups could feel lonely due to a lack of same-age peers, which was often linked to issues understanding one another (Cela & Fokkema, 2017, Dahlberg, 2007, Martin et al., 2014, McInnis & White, 2001, Rönkä et al., 2018, Sawir et al., 2008). Loneliness was also experienced due to a lack of co-ethnic peers in studies of children and older adults who had migrated (Cela & Fokkema, 2017, Kirova-Petrova, 2000, Sawir et al., 2008, Wright-St Clair & Nayar, 2020). Some individuals also experienced loneliness because they lacked like-minded social contacts (Cela & Fokkema, 2017, Tiilikainen & Seppänen, 2017).

## Loneliness relates to a lack of close, meaningful relationships—not superficial connections

*[T]hat you, you miss someone to … to really trust, to really know … (mm) that these are people who’ll be there for you come rain come shine, then you feel lonely.* (Dahlberg, 2007)

Meaningful connections and their absence were central to loneliness for people across the lifespan. It was consistently a lack or loss of meaningful relationships which precipitated loneliness (Barke, 2017, Berguno et al., 2004, Cela & Fokkema, 2017, Dahlberg, 2007, Davidson, 1995, Firmin et al., 2014, Heravi-Karimooi et al., 2010, Herz & Lalander, 2017, Karnick, 2008, Kirova-Petrova, 2000, Kristensen, 1995, Lee et al., 2019, McInnis & White, 2001, Ojembe & Ebe Kalu, 2018, Rew, 2000, Rokach, 1988, Rönkä et al., 2018, Sawir et al., 2008, Smith, 2012, Tiilikainen & Seppänen, 2017, Wright-St Clair & Nayar, 2020). Meaningful relationships involved sharing thoughts and experiences and experiencing intimacy with important others (Cela & Fokkema, 2017, Dahlberg, 2007, Firmin et al., 2014, Graneheim & Lundman, 2010, Hemberg et al., 2021, Heravi-Karimooi et al., 2010, Karnick, 2008, Kirova-Petrova, 2000, Lee et al., 2019, Martin et al., 2014, Rokach, 1988, Rönkä et al., 2018, Sawir et al., 2008, Smith, 2012, Sullivan et al., 2016, Tiilikainen & Seppänen, 2017, Wright-St Clair & Nayar, 2020). Loneliness came about due to a lack of people one really trusted, knew, and was compatible with and understood by. It wasn’t a total lack of relationships that was implicated in loneliness, but a dearth of important connections. Lonely people desired closeness, understanding, and intimacy, as well as relationships where they were authentically engaged and confirmed as a person (Cela & Fokkema, 2017, Dahlberg, 2007, Davidson, 1995, Firmin et al., 2014, Graneheim & Lundman, 2010, Heravi-Karimooi et al., 2010, Kristensen, 1995, Lee et al., 2019, Martin et al., 2014, McInnis & White, 2001, Rokach, 1988, Tiilikainen & Seppänen, 2017, Wright-St Clair & Nayar, 2020).

*I would like to gather those who live here and tell them my story so they will know who I am and who I was …* (Graneheim & Lundman, 2010)

While superficial connections could be sought to temporarily alleviate loneliness, they were not sufficient to prevent it (Cela & Fokkema, 2017, Dahlberg, 2007, Kirova-Petrova, 2000, Kristensen, 1995, McInnis & White, 2001, Ojembe & Ebe Kalu, 2018, Tiilikainen & Seppänen, 2017). Instead, loneliness could be heightened by being with people that one did not choose or did not truly connect with.

## Loneliness involves feelings of disconnection

*[T]here’s something about being lonely that isn’t just about being with someone else. It’s a depth of understanding of your situation.* (Lee et al., 2019)

Loneliness involved feelings of disconnection from other people and the world, with lonely people feeling alone and cut off from others. Loneliness could involve feelings of aloneness, disconnection, not fitting in, not being understood, and lack of fit with surrounding social groups (Barke, 2017, Berguno et al., 2004, Cela & Fokkema, 2017, Dahlberg, 2007, Davidson, 1995, Hemberg et al., 2021, Heravi-Karimooi et al., 2010, Herz & Lalander, 2017, Karnick, 2008, Kirova-Petrova, 2000, Kristensen, 1995, Lee et al., 2019, Martin et al., 2014, Morgan & Burholt, 2020, Rokach, 1988, Rönkä et al., 2018, Sawir et al., 2008, Smith, 2012, Sullivan et al., 2016, Tiilikainen & Seppänen, 2017, Wright-St Clair & Nayar, 2020). It could also relate to a more profound sense of disconnection from the world that could be existential in nature or due to feeling out of step with the wider world (Barke, 2017, Dahlberg, 2007, Davidson, 1995, Kristensen, 1995, Ojembe & Ebe Kalu, 2018, Rokach, 1988, Smith, 2012).

### Loneliness can be eased through connection

Connection with others, whether reaching out to loved ones, exchanging pleasantries, or helping others, could ease loneliness (Barke, 2017, Berguno et al., 2004, Dahlberg, 2007, Davidson, 1995, Firmin et al., 2014, Graneheim & Lundman, 2010, Hemberg et al., 2021, Heravi-Karimooi et al., 2010, Herz & Lalander, 2017, Karnick, 2008, Kirova-Petrova, 2000, Kristensen, 1995, Lee et al., 2019, Martin et al., 2014, McKenna-Plumley et al., 2021, Morgan & Burholt, 2020, Ojembe & Ebe Kalu, 2018, Rew, 2000, Rönkä et al., 2018, Sawir et al., 2008, Smith, 2012, Sullivan et al., 2016, Tiilikainen & Seppänen, 2017, Wright-St Clair & Nayar, 2020).

*Most times, I just walk around my neighbourhood just to exchange pleasantries and have a cordial discussion … that helps me from feeling so lonely.* (Ojembe & Ebe Kalu, 2018)

Connection provided social support and distraction to keep loneliness at bay. When human connection was unavailable, lonely individuals described utilizing connections to nature, animals, a god, soft toy, or forms of social surrogacy such as television and reminiscing to alleviate loneliness (Dahlberg, 2007, Davidson, 1995, Graneheim & Lundman, 2010, Heravi-Karimooi et al., 2010, Herz & Lalander, 2017, Kirova-Petrova, 2000, Kwegyir Tsiboe, 2021, McInnis & White, 2001, Rew, 2000, Smith, 2012, Sullivan et al., 2016, Tiilikainen & Seppänen, 2017).

## Loneliness involves negative interpersonal experiences

*Everyone was calling me names. No matter what I did I couldn’t fit. I was lonely, very lonely*.

(Kirova-Petrova,^2000^)

The experience of loneliness was often related to difficult experiences with other people such as being left out, rejected, and betrayed (Dahlberg, 2007, Davidson, 1995, Firmin et al., 2014, Hemberg et al., 2021, Heravi-Karimooi et al., 2010, Herz & Lalander, 2017, Karnick, 2008, Kirova-Petrova, 2000, Kristensen, 1995, Kwegyir Tsiboe, 2021, Lee et al., 2019, Martin et al., 2014, Rokach, 1988, Rönkä et al., 2018, Smith, 2012, Tiilikainen & Seppänen, 2017, Wright-St Clair & Nayar, 2020). Experiences of conflict were particularly relevant for children and young people. Being bullied at school was implicated in loneliness by children and young adults (Berguno et al., 2004, Kirova-Petrova, 2000, Rönkä et al., 2018), while abuse and lack of respect were mentioned by some older adults (Heravi-Karimooi et al., 2010). Experiences of ostracism and discrimination due to sexuality, race, ethnicity, immigration, and differing from group norms were also salient (Cela & Fokkema, 2017, Kwegyir Tsiboe, 2021, Lee et al., 2019, McInnis & White, 2001, Rokach, 1988, Wright-St Clair & Nayar, 2020).

These negative interpersonal experiences were linked with feelings that lonely individuals had about themselves in relation to others. These included feeling that no one cared about or liked them, that they were different, invisible, and alienated from others, and that they were forgotten and abandoned (Barke, 2017, Dahlberg, 2007, Davidson, 1995, Graneheim & Lundman, 2010, Hemberg et al., 2021, Heravi-Karimooi et al., 2010, Herz & Lalander, 2017, Karnick, 2008, Kirova-Petrova, 2000, Kristensen, 1995, Lee et al., 2019, Martin et al., 2014, McInnis & White, 2001, Rew, 2000, Rokach, 1988, Rönkä et al., 2018, Sawir et al., 2008, Smith, 2012, Sullivan et al., 2016, Tiilikainen & Seppänen, 2017).

*Well I tried a few times but you know kids don’t like me, they just like kind of ignore me.* (Martin et al., 2014)

While these feelings were less strictly linked to specific occurrences and could potentially represent inaccurate perceptions, they were related to interpersonal experiences that left the person feeling unwanted, inferior, and alone.

## Loneliness involves social comparison

*[W]hen seeing that everyone else find their groups and friends and have fun … And seeing that they enjoy their groups, one feels extra lonely then.* (Hemberg et al., 2021)

Loneliness often resulted from social comparison. These comparisons were drawn by the lonely person between themselves and others, for example when noticing others who seemed more socially active or satisfied (Barke, 2017, Dahlberg, 2007, Davidson, 1995, Firmin et al., 2014, Hemberg et al., 2021, Karnick, 2008, Lee et al., 2019, McInnis & White, 2001, Rönkä et al., 2018, Tiilikainen & Seppänen, 2017). Loneliness could also arise when comparing one’s current social situations to a previous more socially satisfying period (Cela & Fokkema, 2017, Firmin et al., 2014, Heravi-Karimooi et al., 2010, Kirova-Petrova, 2000, Lee et al., 2019, Morgan & Burholt, 2020, Tiilikainen & Seppänen, 2017). Unmet social norms could also provoke these feelings, for example when one was not socializing at commonly social times like weekend nights or holidays (Dahlberg, 2007, Lee et al., 2019, Rokach, 1988, Rönkä et al., 2018, Sullivan et al., 2016).

Loneliness could also emerge when people made comparisons with unrealized possibilities and when social expectations were not met (Dahlberg, 2007, Lee et al., 2019, Morgan & Burholt, 2020, Rokach, 1988, Rönkä et al., 2018, Sawir et al., 2008, Tiilikainen & Seppänen, 2017). This happened when reality did not align with prior expectations that others would be there for them or share responsibility. Loneliness existed in relation to a context of fellowship that was missing.

### Personal contextual aspects of the experience

Six descriptive themes represent aspects of the loneliness experience related to personal context.

## Loneliness is connected to, but separate from, aloneness, isolation, and solitude

*I have a huge amount of friends and I am a prime example of how immensely lonely one can be in the midst of a friendship group.* (Rönkä et al., 2018)

Loneliness was consistently connected with isolation (Barke, 2017, Berguno et al., 2004, Cela & Fokkema, 2017, Dahlberg, 2007, Davidson, 1995, Firmin et al., 2014, Graneheim & Lundman, 2010, Hemberg et al., 2021, Heravi-Karimooi et al., 2010, Karnick, 2008, Kirova-Petrova, 2000, Kristensen, 1995, Kwegyir Tsiboe, 2021, Lee et al., 2019, Martin et al., 2014, McInnis & White, 2001, McKenna-Plumley et al., 2021, Ojembe & Ebe Kalu, 2018, Park et al., 2019, Rew, 2000, Rokach, 1988, Rönkä et al., 2018, Sawir et al., 2008, Smith, 2012, Sullivan et al., 2016, Tiilikainen & Seppänen, 2017, Wright-St Clair & Nayar, 2020). People reported feeling lonely due to having no, little, or reduced social contact. In some cases, loneliness occurred due to a lack of social support, instrumental support, or people to do things with (Barke, 2017, Berguno et al., 2004, Cela & Fokkema, 2017, Davidson, 1995, Firmin et al., 2014, Hemberg et al., 2021, Heravi-Karimooi et al., 2010, Herz & Lalander, 2017, Karnick, 2008, Kirova-Petrova, 2000, Kristensen, 1995, Lee et al., 2019, McInnis & White, 2001, McKenna-Plumley et al., 2021, Morgan & Burholt, 2020, Ojembe & Ebe Kalu, 2018, Sawir et al., 2008, Smith, 2012, Tiilikainen & Seppänen, 2017).

*I didn’t have anyone to talk to, that was the thing. It was really hard because I couldn’t have contact with anybody.* (Sawir et al., 2008)

Loneliness existed in relation to the social world and some people suggested that having more contacts could protect against loneliness. However, loneliness was also experienced by people who were socially embedded, indicating the separation of objective isolation and the subjective feeling of loneliness.

Being alone, particularly when there was nothing to do, was commonly mentioned (Cela & Fokkema, 2017, Dahlberg, 2007, Karnick, 2008, Kristensen, 1995, Kwegyir Tsiboe, 2021, Martin et al., 2014, McInnis & White, 2001, Park et al., 2019, Rew, 2000, Rönkä et al., 2018, Smith, 2012, Sullivan et al., 2016, Tiilikainen & Seppänen, 2017). Loneliness could also be a response to existential isolation, when one experienced a fundamental sense of being truly alone and in another world from others (Dahlberg, 2007, Davidson, 1995, Graneheim & Lundman, 2010, Hemberg et al., 2021, Heravi-Karimooi et al., 2010, Kristensen, 1995, Lee et al., 2019, Rokach, 1988, Rönkä et al., 2018, Sullivan et al., 2016). Nevertheless, people consistently described loneliness when they were not alone (Cela & Fokkema, 2017, Dahlberg, 2007, Davidson, 1995, Heravi-Karimooi et al., 2010, Herz & Lalander, 2017, Kirova-Petrova, 2000, Kristensen, 1995, Lee et al., 2019, McInnis & White, 2001, McKenna-Plumley et al., 2021, Morgan & Burholt, 2020, Ojembe & Ebe Kalu, 2018, Rokach, 1988, Rönkä et al., 2018, Sawir et al., 2008, Smith, 2012, Sullivan et al., 2016, Tiilikainen & Seppänen, 2017, Wright-St Clair & Nayar, 2020), and many reported being alone without feeling lonely (Davidson, 1995, Graneheim & Lundman, 2010, Hemberg et al., 2021, Herz & Lalander, 2017, Kristensen, 1995, Sullivan et al., 2016).

In describing loneliness, solitude (or a chosen, enjoyed aloneness) was mentioned by some (Graneheim & Lundman, 2010, Hemberg et al., 2021, Karnick, 2008, Kristensen, 1995, Martin et al., 2014, McInnis & White, 2001, Sawir et al., 2008, Sullivan et al., 2016). When alone, people could experience rest, freedom, and peace, and being alone was preferable if one needed to recuperate or did not want to socialize. However, the valence of aloneness could vary and too much time alone could be negative.

Loneliness was conflated with aloneness and solitude in some cases (Dahlberg, 2007, Karnick, 2008, Martin et al., 2014, McInnis & White, 2001, Rew, 2000). Some studies referred to “voluntary” loneliness, which may otherwise be described as solitude. Some people also described aloneness when asked about loneliness. This indicates that there may be room in descriptions of loneliness for a positive interpretation linked to chosen aloneness, which was described by some studies (see theme 1.2), or that the distinctions used in research between these constructs are sometimes used more loosely in lived descriptions of loneliness.

### Loneliness involves a lack of control

A common feature was lack of control over the conditions surrounding loneliness (Dahlberg, 2007, Davidson, 1995, Hemberg et al., 2021, Heravi-Karimooi et al., 2010, Herz & Lalander, 2017, Kirova-Petrova, 2000, Kristensen, 1995, Lee et al., 2019, McKenna-Plumley et al., 2021, Ojembe & Ebe Kalu, 2018, Rokach, 1988). Lonely people could perceive little or no control over social contacts and loneliness was experienced when agency was removed and barriers to connection like language or physical restrictions were present (Cela & Fokkema, 2017, Herz & Lalander, 2017, Kirova-Petrova, 2000, Lee et al., 2019, Park et al., 2019, Sawir et al., 2008, Smith, 2012, Wright-St Clair & Nayar, 2020).

## Loneliness is precipitated by life experiences and transitions

*Last year was different because I was new to this campus, like, it was a new experience for me. So, I just had a lot of new experiences, so those were the lonely times.* (Firmin et al., 2014)

Life experiences and particularly transitions were consistently raised as precipitating factors for loneliness. Adverse life experiences were often linked to loneliness, with lonely individuals mentioning difficulties related to ageing (Heravi-Karimooi et al., 2010, Kwegyir Tsiboe, 2021, McInnis & White, 2001, Ojembe & Ebe Kalu, 2018, Sullivan et al., 2016, Wright-St Clair & Nayar, 2020), childhood adversity (Rönkä et al., 2018, Tiilikainen & Seppänen, 2017), abuse (Heravi-Karimooi et al., 2010, Rönkä et al., 2018), and identity struggles (Firmin et al., 2014, Morgan & Burholt, 2020). While loneliness could be precipitated by specific experiences, it could also follow an accumulation of experiences across the past, present, and unknowable future (Barke, 2017, Cela & Fokkema, 2017, Dahlberg, 2007, Davidson, 1995, Graneheim & Lundman, 2010, Heravi-Karimooi et al., 2010, Herz & Lalander, 2017, Karnick, 2008, Morgan & Burholt, 2020, Rew, 2000, Rokach, 1988, Rönkä et al., 2018, Sullivan et al., 2016, Tiilikainen & Seppänen, 2017).

### Loneliness during social transitions

Loneliness was commonly experienced during transitions which altered the social world. Bereavement, particularly of a spouse, commonly precipitated loneliness (Barke, 2017, Dahlberg, 2007, Davidson, 1995, Graneheim & Lundman, 2010, Heravi-Karimooi et al., 2010, Kwegyir Tsiboe, 2021, McInnis & White, 2001, Morgan & Burholt, 2020, Ojembe & Ebe Kalu, 2018, Rew, 2000, Rokach, 1988, Smith, 2012, Sullivan et al., 2016, Tiilikainen & Seppänen, 2017). Grieving was lonely, as was reorienting to the world without a partner.

*I’ve got everything but I haven’t got enough […] If you love somebody for that many years, it’s a very lonely life.* (Sullivan et al., 2016)

Separation from loved ones through divorce or separation, conflict, or geographical distance could also intensify loneliness (Berguno et al., 2004, Dahlberg, 2007, Firmin et al., 2014, Hemberg et al., 2021, Heravi-Karimooi et al., 2010, Herz & Lalander, 2017, Lee et al., 2019, Martin et al., 2014, McInnis & White, 2001, Morgan & Burholt, 2020, Rew, 2000, Rokach, 1988, Sawir et al., 2008). This was also described in situations of retirement (Barke, 2017, Kwegyir Tsiboe, 2021, Ojembe & Ebe Kalu, 2018, Smith, 2012, Sullivan et al., 2016), due to the subsequent identity and social shift, and relocation (Cela & Fokkema, 2017, Davidson, 1995, Firmin et al., 2014, Herz & Lalander, 2017, Kristensen, 1995, Kwegyir Tsiboe, 2021, Martin et al., 2014, Morgan & Burholt, 2020, Rokach, 1988, Rönkä et al., 2018, Sawir et al., 2008, Sullivan et al., 2016, Tiilikainen & Seppänen, 2017, Wright-St Clair & Nayar, 2020). For others, new experiences such as becoming a mother (Lee et al., 2019) or entering COVID−19 lockdown (McKenna-Plumley et al., 2021) brought about social changes that impacted loneliness. Various experiences that increased isolation or otherwise impacted the social world generated loneliness, some of which, such as retirement and becoming widowed, were particularly relevant at certain stages of the lifespan.

## Loneliness fluctuates in duration, intensity, and type

*Some points in my life now I feel very, very lonely, and there’s some point that I don’t feel lonely at all.* (Rew, 2000)

Loneliness was depicted as a trait for some and could be experienced as common or constant (Barke, 2017, Berguno et al., 2004, Cela & Fokkema, 2017, Davidson, 1995, Kristensen, 1995, Morgan & Burholt, 2020, Rew, 2000, Rönkä et al., 2018, Sawir et al., 2008, Sullivan et al., 2016, Tiilikainen & Seppänen, 2017). For others, loneliness was transient and fluctuating (Cela & Fokkema, 2017, Firmin et al., 2014, Herz & Lalander, 2017, Martin et al., 2014, McKenna-Plumley et al., 2021, Morgan & Burholt, 2020, Rönkä et al., 2018). People could feel lonely more severely or subtly and recognize times when they felt more or less lonely, not just lonely or not-lonely (Barke, 2017, Cela & Fokkema, 2017, Dahlberg, 2007, Davidson, 1995, Firmin et al., 2014, Rew, 2000, Rönkä et al., 2018, Sawir et al., 2008, Sullivan et al., 2016). Although sometimes labelled as common or inevitable (Heravi-Karimooi et al., 2010, McInnis & White, 2001, Ojembe & Ebe Kalu, 2018, Sawir et al., 2008), loneliness was difficult to describe and experiences varied (Dahlberg, 2007, Davidson, 1995, Firmin et al., 2014, Rönkä et al., 2018, Sawir et al., 2008, Sullivan et al., 2016). It could connect to different factors and take different forms, such as a specific type of loneliness or a worst kind of loneliness. Loneliness was not necessarily one-dimensional.

Loneliness also sometimes appeared as a process, following from discrete or cumulative life events and requiring resolution (Karnick, 2008, Morgan & Burholt, 2020, Rönkä et al., 2018, Sullivan et al., 2016). It was also related to the ageing process; some older people anticipated loneliness in later life and some participants mentioned that social priorities could change with age such that lacking different types of relationships might precipitate loneliness at different life stages (Barke, 2017, Graneheim & Lundman, 2010, Hemberg et al., 2021, Heravi-Karimooi et al., 2010, Kwegyir Tsiboe, 2021, Martin et al., 2014, McInnis & White, 2001, Ojembe & Ebe Kalu, 2018, Rönkä et al., 2018, Sawir et al., 2008, Smith, 2012, Sullivan et al., 2016, Tiilikainen & Seppänen, 2017). Life experience was described as instrumental for coping with loneliness.

## Loneliness can be grounded in specific contexts

*“I just get loneliness at night … more at night.”* (Rew, 2000)

While loneliness was sometimes positioned as a trait, it could also emerge or intensify in specific situations. Loneliness could occur in free time or leisure periods and subside when occupied (Berguno et al., 2004, Cela & Fokkema, 2017, Hemberg et al., 2021, Karnick, 2008, Kirova-Petrova, 2000, Kristensen, 1995, Kwegyir Tsiboe, 2021, McInnis & White, 2001, Morgan & Burholt, 2020, Sullivan et al., 2016, Tiilikainen & Seppänen, 2017). It could also emerge in difficult periods, on bad days and unhappy times (Dahlberg, 2007, Firmin et al., 2014, Rokach, 1988, Sawir et al., 2008). Specific periods such as evenings, night-time, weekends, and winter were described as key periods for loneliness (Barke, 2017, Davidson, 1995, Karnick, 2008, Lee et al., 2019, McInnis & White, 2001, Rew, 2000, Rönkä et al., 2018, Smith, 2012, Sullivan et al., 2016, Tiilikainen & Seppänen, 2017). Special occasions like birthdays and holidays like Christmas were also times when loneliness could peak due to actual or perceived isolation (Firmin et al., 2014, Hemberg et al., 2021, Rew, 2000, Rönkä et al., 2018, Sawir et al., 2008).

*New Year and Midsummer are … [holidays] they’re the worst for me, school breaks ups etc. Because these are what I call “Have fun with your friends – If you have any” holidays …* (Hemberg et al., 2021)

Physical spaces such as isolated geographical areas, home, school, and foreign countries could also be lonely. Being in a foreign culture could be particularly lonely due to the lack of social contacts, different social and cultural norms, and unfamiliarity (Cela & Fokkema, 2017, Herz & Lalander, 2017, Kirova-Petrova, 2000, Park et al., 2019, Sawir et al., 2008, Wright-St Clair & Nayar, 2020). Homesickness was linked to loneliness by some (Cela & Fokkema, 2017, Dahlberg, 2007, Rew, 2000, Sawir et al., 2008) and people could feel lonely in specific physical contexts (Park et al., 2019, Rönkä et al., 2018, Tiilikainen & Seppänen, 2017), such as at work, or in relation to specific relationships (Cela & Fokkema, 2017, Dahlberg, 2007, Lee et al., 2019, Sullivan et al., 2016), such as close companions.

## Loneliness is impacted by physical and mental health challenges

*That’s another thing that keeps me so lonely … Assuming that my legs were as strong as it was before, I would have being going out myself and attend functions without waiting for visitor, friends or even family to visit me …* (Ojembe & Ebe Kalu, 2018)

Loneliness could arise or intensify due to poor physical health, functional decline, physical disability, and mental health issues (Cela & Fokkema, 2017, Dahlberg, 2007, Graneheim & Lundman, 2010, Hemberg et al., 2021, Heravi-Karimooi et al., 2010, Kwegyir Tsiboe, 2021, Lee et al., 2019, McInnis & White, 2001, Morgan & Burholt, 2020, Ojembe & Ebe Kalu, 2018, Park et al., 2019, Rew, 2000, Rokach, 1988, Rönkä et al., 2018, Sawir et al., 2008, Smith, 2012, Sullivan et al., 2016, Tiilikainen & Seppänen, 2017, Wright-St Clair & Nayar, 2020). Health and loneliness interacted in various ways, but perhaps most centrally, poor health limited independence and could make it difficult to socialize (Graneheim & Lundman, 2010, Heravi-Karimooi et al., 2010, Lee et al., 2019, McInnis & White, 2001, Morgan & Burholt, 2020, Ojembe & Ebe Kalu, 2018, Smith, 2012, Tiilikainen & Seppänen, 2017). Unmet needs, limited social interactions, and reduced agency were implicated in loneliness. Physical health issues and disability could also provoke hopelessness and a sense that one was different or conspicuous. Loneliness was also intertwined with mental health. It could occur alongside or due to depression and other mental health issues (Barke, 2017, Cela & Fokkema, 2017, Hemberg et al., 2021, Heravi-Karimooi et al., 2010, Kristensen, 1995, McKenna-Plumley et al., 2021, Rönkä et al., 2018, Sawir et al., 2008), and experiences of depression and anxiety could intensify loneliness.

*“I was getting really depressed and lonely,”* (Sawir et al., 2008)

Discussion around physical health and loneliness featured prominently in studies of older adults (e.g., Heravi-Karimooi et al., 2010, Kwegyir Tsiboe, 2021, Morgan & Burholt, 2020, Ojembe & Ebe Kalu, 2018, Smith, 2012), who often mentioned physical declines, but were also present in some younger people who had experienced ill health (Hemberg et al., 2021, Rönkä et al., 2018).

### Loneliness has a physical dimension

Relatedly, loneliness appeared to have a physical dimension, being experienced as embodied (Dahlberg, 2007, Firmin et al., 2014, Rokach, 1988, Rönkä et al., 2018, Sullivan et al., 2016, Wright-St Clair & Nayar, 2020) and accompanied by physical symptoms in some cases (Dahlberg, 2007, Hemberg et al., 2021, Kirova-Petrova, 2000, McInnis & White, 2001, Rokach, 1988, Rönkä et al., 2018). Loneliness could be painful and weigh individuals down in a metaphorical and physical sense. Physical symptoms included impaired sensory perception, stiffness, tightness in the chest, numbness, aches, changes in appetite, and feelings of being in fight or flight.

## Loneliness is affected by the socio-political landscape

*”[T]hey [children] have their own financial problems, we don’t want to ask them for money for a bus ticket to go downtown. Having friends means going to a bar, taking a coffee, one time you pay for them and next time they pay for you. But without money what can we do? Where can we go?”* (Cela & Fokkema, 2017)

Loneliness was impacted by social and cultural norms as well as the availability of resources and services. One could be lonely in a foreign culture, where ways of socializing were unfamiliar, or in their own culture due to societal changes and lack of fit (Barke, 2017, Heravi-Karimooi et al., 2010, Kwegyir Tsiboe, 2021, Ojembe & Ebe Kalu, 2018, Rönkä et al., 2018, Tiilikainen & Seppänen, 2017). Heterogendered norms could impact the types of relationships that were available and seen as valuable and affect willingness to talk about and accept loneliness (Cela & Fokkema, 2017, Heravi-Karimooi et al., 2010, Kwegyir Tsiboe, 2021, Lee et al., 2019, McInnis & White, 2001, Morgan & Burholt, 2020, Rönkä et al., 2018, Sawir et al., 2008, Smith, 2012). Religious and cultural norms around social connection were also mentioned (Barke, 2017, Cela & Fokkema, 2017, Kwegyir Tsiboe, 2021, Rokach, 1988, Rönkä et al., 2018, Sullivan et al., 2016, Wright-St Clair & Nayar, 2020).

Discrimination could also impact loneliness. Some studies described ageism, racism, homophobia, and discrimination based on ethnicity (Cela & Fokkema, 2017, Kwegyir Tsiboe, 2021, Sullivan et al., 2016, Wright-St Clair & Nayar, 2020). The related social rejection and abuse impacted social networks with subsequent impacts on loneliness.

Objective barriers to connection were also implicated. A lack of money, time, transport, meeting places, and services were relevant (Cela & Fokkema, 2017, Dahlberg, 2007, Heravi-Karimooi et al., 2010, Herz & Lalander, 2017, Kwegyir Tsiboe, 2021, Ojembe & Ebe Kalu, 2018, Park et al., 2019, Rönkä et al., 2018, Sawir et al., 2008, Smith, 2012, Sullivan et al., 2016, Tiilikainen & Seppänen, 2017, Wright-St Clair & Nayar, 2020). Loneliness occurred when people lacked money to engage in social activities, when people had to work so much that socializing was not possible, when transport was unavailable or too expensive, and when meeting places were unavailable.

*[I]nadequate local public transportation system makes it difficult to get access to hospitals, doctors, relatives, religious centres and cultural centres.* (Heravi-Karimooi et al., 2010)

A lack of government provision and its resultant impact on the ability to connect were relevant. A lack of community and recreational activities was implicated in loneliness (Heravi-Karimooi et al., 2010, Kwegyir Tsiboe, 2021, Ojembe & Ebe Kalu, 2018, Park et al., 2019). For others, policy had direct implications, such as unaccompanied young migrants whose housing regulations and changing guardianship made it difficult to sustain relationships (Herz & Lalander, 2017, Kwegyir Tsiboe, 2021, Lee et al., 2019, McKenna-Plumley et al., 2021, Ojembe & Ebe Kalu, 2018, Park et al., 2019). In one study, older adults invoked the better financial support available in New Zealand as worthy compensation for the isolation they experienced there as migrants (Park et al., 2019). Inadequate government services were directly invoked by some (Kwegyir Tsiboe, 2021, Ojembe & Ebe Kalu, 2018). Social and political barriers to connection including discrimination, lack of fit with dominant norms, and inadequate provision of resources and services influenced loneliness.

### Analytical themes

Three analytical themes were developed from the descriptive themes to address the research question (“How do people describe their experiences of loneliness?”) at a holistic level.

## Loneliness is both psychological and contextual

Loneliness appears to be a psychological state that is also inherently related to one’s interpersonal and personal context, existing in relation to the physical, social, relational, cultural, and political context of the lonely person. While loneliness is intrinsically of the mind—an aversive emotional experience with cognitive and perceptual features—it is not isolated from the interpersonal and societal conditions of the lonely person. It is necessary to stress the separation of objective conditions of social isolation and the subjective experience of loneliness, but it appears that loneliness is still intimately connected to one’s perceptions and experiences of the social world around them, as well as the conditions of their life. There was an overarching sense that personal psychology and the contextual aspects of one’s life were central to descriptions of loneliness experiences. Feelings of sadness, cognitive appraisals, and social comparisons were all features of loneliness, but loneliness arose in relation to circumstances such as social transitions, physical decline, discrimination, and lack of resources which could be related to specific life stages. Loneliness is not experienced in a vacuum despite its subjective nature; although it is characterized by psychological features including a set of feelings and perceptions, it is also grounded in objective circumstances.

## Loneliness centres on feelings of meaningful connection and painful disconnection

Although being alone and isolated are related to loneliness, it is subjective feelings of connection and the feeling of aloneness that are central to the experience. Loneliness involves feelings of disconnection which are aversive, unwanted, and beyond one’s control, and is experienced in relation to important, meaningful relationships with other people which are felt to be lost, lacking, or absent. When people describe loneliness, they talk about important connections with other people which lessen loneliness or could if they were available. These descriptions involved discussion of faraway partners, broken friendships, busy children, and deceased spouses, as well as meaningful connections which were altogether lacking. It is one’s sense that they are disconnected from important others which brings about loneliness rather than just objective, superficial aspects of the social world. Loneliness is situated against a contrasting backdrop of meaningful connection.

## Loneliness can exist in a general, pervasive sense or can relate to specific other people or relationship types

Some people experience loneliness due to general dissatisfaction with their relationships or a feeling of overarching disconnection from the world. However, this synthesis underlined that in many cases, loneliness is felt in relation to specific people or types of relationships. Rather than a holistic sense of isolation, it can be due to a lack of friends or a sense that likeminded people are missing from one’s life. A person who is generally satisfied socially may experience loneliness when a spouse passes away or a friendship is lost. While loneliness appears to generally share some common features—a sense of aversiveness, a feeling of disconnection—it is important to bear in mind that loneliness can occur due to a multitude of factors, from the death of a spouse to the lack of same-age friends to a feeling of disconnection from society. In this respect, loneliness appears to be a multidimensional experience which can stem from various causes and require diverse, life stage-sensitive, and individually-grounded understandings.

### Sensitivity analysis

Sensitivity analysis was carried out to assess the impact of lower-quality studies and specific age groups on the synthesis. Impact was assessed by considering whether any descriptive themes became less well-evidenced (for example, less rich or thick) when these studies were removed.

### Did “lower-quality” studies impact the synthesis?

Study quality may not be best reflected through quality appraisal checklists (for example, if a study is reported inadequately due to word count limitations). However, for the purpose of sensitivity analysis, a cut-off point of 4 or below out of 10 on the JBI checklist was used to delineate “lower-quality” studies, as this was the level at which fewer than half of the criteria were evidenced. Five studies scored 4 or below, although in some cases it appeared that this was due to reporting.

None of the 15 descriptive themes depended exclusively on the lower-quality studies and all were contributed to by these studies. Some aspects related to migration (for example, that being in a foreign culture was lonely, that a lack of co-ethnic meeting spaces was missed, and that migration was linked to loneliness) were less thick due to the removal of studies by Cela and Fokkema (2017) and Sawir and colleagues (2008), but these elements and the overarching themes remained. No theme was largely diminished by the removal of lower-quality studies, perhaps due to the large volume of data or because of the imperfect relationship between study quality and formal quality appraisal. Other research has similarly noted a lack of impact from removing low-quality studies (Carroll & Booth, 2015).

### Are any themes specific to particular age groups?

Studies which included only participants within a specific age group were excluded one by one to assess the impact of specific age groups (children [0–15 years], young adults [16–29 years], and older adults [60+]) on the synthesis. No studies included only middle-aged adults (30–59 years), so no analysis focuses on this group.

#### Children

Two themes were not contributed to by the five child studies, namely “Loneliness is affected by the socio-political landscape” and “Loneliness is impacted by personality and identity”, indicating that these aspects of loneliness are more specific to or more recognized by adults. Studies of children also contributed minimally to themes around social comparison, physical and mental health challenges, and life experiences and transitions (possibly because children have had less time to accumulate transitions and experiences which are relevant to loneliness). None of the descriptive themes depended exclusively on child studies, although aspects related to being left out were less prevalent when these studies were removed.

#### Young adults

None of the descriptive themes depended exclusively on the two studies including only young adult participants and all themes were contributed to by the young adult studies. While no theme depended exclusively on these studies, the studies contributed largely to the aspect of relocation in relation to loneliness. Additionally, data which directly described heterogendered norms by name were attributable entirely to one young adult study (Rönkä et al., 2018). However, data relating the relevance of heterogendered norms and relocation to loneliness were still present from other studies.

#### Older adults

None of the 15 descriptive themes depended exclusively on the 11 studies including only older adult participants and all themes were contributed to by the older adult studies. Most themes were impacted by the removal of 11 of the 29 studies, but most notably, several aspects of the theme “Loneliness is affected by the socio-political landscape” were lessened or erased. The sense that activities could manage loneliness as well as codes around the need for government involvement, cultural norms around ageing, ageism, and lack of access to services were entirely lost. Similarly, codes around inadequate government services, lack of transport, and societal change were depleted. Another large impact was in the theme “Loneliness is impacted by physical and mental health challenges”, where codes around bodily decline were altogether removed and aspects of lack of independence and disability were largely impacted. Poor health remained as a major factor but 10 of the 11 older adult studies had contributed to it. Similarly, in terms of life transitions, codes around bereavement, reduced opportunities for interaction, and separation from one’s partner were largely impacted.

## Discussion

The current study used systematic review and thematic synthesis of 29 studies to understand how loneliness is subjectively experienced across the lifespan, enhancing our understanding of loneliness by providing a novel, fine-grained synthesis of lived experiences from 1,321 participants from 7 to 103 years old. The synthesis found that loneliness is a multifaceted experience which depends both on personal psychology and one’s personal, social, political, and cultural context, that it hinges on the distinction between feelings of meaningful connection and painful disconnection, and that it can be felt in a general, pervasive sense or in relation to deficits in specific relationships. Loneliness experiences centred around psychological features, interpersonal aspects, and elements of one’s personal context.

### Psychological features of the loneliness experience

Loneliness appears to be intrinsically psychological despite its relationship to various contextual factors. Many studies have described loneliness as a distressing feeling (Hawkley & Cacioppo, 2010, Lam et al., 2021, Perlman & Peplau, 1981) but the present synthesis expands on this to illuminate the emotional, cognitive, and perceptual components of loneliness. Loneliness feels a certain way—sad, empty, and scary—and is cyclically related to other feelings like boredom. The synthesis uncovered emotional nuances of the loneliness experience which extend beyond simple distress, supporting Bound Alberti’s (2018) conceptualization of loneliness as an emotion cluster, referring to its potential to include multiple separate and potentially competing emotions. Loneliness also included cognitive components including negative appraisals of oneself and one’s social world. Cognitive mechanisms are clearly influential to loneliness; research with adolescents indicates that perceived, rather than actual, social acceptance mediates the relationship between self-esteem and loneliness (Vanhalst et al., 2013). Similarly, loneliness appears to be impacted by social comparison with other people, previous circumstances, or imagined alternatives. This finding is consistent with other qualitative work on loneliness trajectories (Fardghassemi et al., 2022, Morgan & Burholt, 2022) but social comparison is relatively absent from loneliness research, despite the discrepancy between desired and actual relationships being central to the most common definition of loneliness (Perlman & Peplau, 1981). The predominant cognitive theory of loneliness describes how individuals judge their social relationships against an internal desired standard (Perlman & Peplau, 1981) but these findings suggest that external comparisons also play an important role in loneliness.

Perceptual components were another psychological aspect evidenced in this synthesis. Loneliness was described as feeling cold and impacting time perception. Experimental work has demonstrated that experiences of exclusion can lower skin temperature (Ijzerman et al., 2012), which may explain why loneliness feel cold. Social exclusion and rejection also impact light and time perception, with excluded individuals perceiving higher levels of darkness (Pfundmair et al., 2019) and overestimating time intervals (Twenge et al., 2003), although this synthesis also noted experiences of time passing quickly and stopping. However, research in this area has focused on experimentally manipulated exclusion rather than subjective ratings of loneliness. While cognitive and affective elements are often described in definitions of loneliness, this synthesis suggests that perceptual elements and social comparison warrant further attention. These findings stress the importance of understanding loneliness as an experience that is characterized centrally by psychological components, despite the relevance of objective contextual circumstances.

### Contextual features of the loneliness experience

The relevance of context was clear. Loneliness was impacted by a person’s physical context (e.g., location, time of day), interpersonal and personal context (e.g., relational factors, life events), and socio-political context (e.g., social norms and service provision). Studies using ecological momentary assessment corroborate this finding, indicating that momentary changes in physical context such as being alone are associated with loneliness (Compernolle et al., 2021, Compernolle et al., 2022). Personal context was also pertinent, as loneliness was described in reference to life transitions such as bereavement, which appeared to be particularly impactful when they increased isolation, which has also been found in recent experimental research (Evans et al., 2022). These transitions are not equally distributed across the life course (for example, educational transitions are more common in younger adulthood and retirement in later life), meaning that the contextual influences which precipitate loneliness may be unevenly distributed. This may account for the differing prevalence of loneliness at different life stages (Mund et al., 2020, Victor & Yang, 2012). Indeed, Hawkley and colleagues (2022) found that age differences in life experiences and resources appear to account for age differences in loneliness, rather than age itself. Poor health and functional decline were other personal contextual factors which precipitated loneliness, particularly for older adults, partly due to their deleterious impact on social independence. The relationship between loneliness and poor health is well established (e.g., Hawkley & Cacioppo, 2010) and it is notable that it was a prominent finding of this study given that studies of specific clinical groups were not included.

Importantly, the wider socio-political context also impacted experiences of loneliness. Inadequate provision of services and resources such as community activities, money, and transport were implicated. The lack of government provision was directly raised by some. This finding indicates that policy seeking to ameliorate loneliness should consider the availability of resources and public services, rather than purely social and personal factors. Socioeconomic factors are known predictors of loneliness (Algren et al., 2020, L. Dahlberg & McKee, 2014) and this synthesis shows that they are also subjectively identified as relevant. Wider socio-political factors such as discrimination were also implicated. Discrimination is quantitatively associated with loneliness (Priest et al., 2017; Sutin et al., 2015; Świtaj et al., 2015) and in the present synthesis it appeared that loneliness came about due both to the negative feelings stemming from discrimination and its impact on social networks. It was clear from the synthesis that loneliness could fluctuate in presence and intensity and addressing structural and social drivers may be a key way of ameliorating the experience of loneliness. Research, policy, and theory should take into account that loneliness is not divorced from the lonely person’s physical, personal, and socio-political context.

### Conflating and distinguishing between loneliness and related constructs

The connection between loneliness and related phenomena is also relevant. In the included studies, people who had experienced loneliness often conflated it with phenomena like aloneness, isolation, and solitude. While it is separable from these experiences, loneliness was discussed alongside objective isolation from social connections, time spent alone, and pleasant experiences of solitude. The synthesis supported common conceptualizations of loneliness as aversive, and the emotional, cognitive, and perceptual components of loneliness were generally negative. However, the synthesis evidenced the potential for positive outcomes or interpretations. The experience of loneliness could provide the potential for growth and calm. While some studies referred to loneliness itself as potentially positive, this generally appeared to constitute a voluntary state of aloneness akin to *Einsamkeit* (de Jong Gierveld, 1998), looking on the bright side, or a mechanism similar to post-traumatic growth (Tedeschi & Calhoun, 2004), where lonely people can in some circumstances cope and accommodate the experience. The potential for positive, or at least non-catastrophic, interpretations of loneliness has been touched upon in recent feminist approaches to the topic (Magnet & Orr, 2022, Wilkinson, 2022) and from a philosophical standpoint, with Moustakas (1961) suggesting that existential loneliness can lead to self-growth, but has been generally overlooked in quantitative empirical literature.

The predominant curative approach to loneliness may obscure the potential for positive outcomes and scientific exploration into them. However, it appeared that the positive interpretation of loneliness identified in the review may also be linked to the common conflation of loneliness with aloneness, isolation, and solitude. While distinction between these constructs is important to capture the subjectivity and specificity of loneliness and its generally negative expression (Hauge & Kirkevold, 2010), it may be productive for research to consider the potential for positive growth from loneliness. Moreover, it is important to note that lonely people may not differentiate as strictly between these phenomena as researchers or practitioners.

Discussions of loneliness included some overlap with related phenomena, but it appeared that the key component which distinguished loneliness was the subjective feeling of disconnection in response to a lost, imagined, or desired inverse of meaningful connection. People could experience loneliness when isolated or momentarily alone, but loneliness was not a necessary outcome of these circumstances. Instead, loneliness arises when people feel that they are disconnected, separated, misunderstood, or lacking important connections. A lack of control over the amount or kind of connections that were available was also implicated. The importance of satisfying relationships, rather than just the presence of relationships, has been referred to in the literature (Coyle & Dugan, 2012, Heinrich & Gullone, 2006, Ho et al., 2023, Perlman & Peplau, 1998). This review indicates that satisfying relationships are those which are meaningful, in that they allow intimacy and understanding with important others. Loneliness policy has tended to address relationships in a general sense, encouraging connections broadly, and although meaningful social relationships are discussed in emerging policy from the Campaign to End Loneliness (2020, 2021) and Ending Loneliness Together (2020), what constitutes a meaningful relationship should be operationalized beyond “relationship quality” given that this synthesis indicates that the perception of a lack of meaningful relationships is a central feature of loneliness. The Ending Loneliness Together policy aim to create a Meaningful Relationship Framework (2020) is encouraging to this end.

### Loneliness in relation to specific relationship deficits

Loneliness is often depicted as a general feeling related to overall isolation, but the present findings emphasize that the perceived lack or inadequacy of specific relationships or relationship types, such as having no close friends, the loss of a spouse, or lack of co-ethnic social network, could also cause loneliness. This aligns with Weiss’s (1973) social deficit approach to loneliness which proposes two different types of loneliness based on specific social deficits: emotional loneliness results from the lack of satisfying intimate relationships, while social loneliness occurs when one feels that they lack a sufficient social network. These relationship types broadly fulfil different needs, with intimate relationships satisfying attachment needs and social networks satisfying the need for integration (DiTommaso & Spinner, 1997, Weiss, 1973). Indeed, the current study indicates that the lack of specific types of relationships, such as those which fulfil roles like romantic partner or characteristics like shared culture or age, can provoke loneliness which is not eased by the availability of other connections. This suggests that a multidimensional typology of loneliness, like the approach proposed by Weiss (1973), should be considered alongside more prevalent theoretical approaches such as cognitive (Perlman & Peplau, 1981) and evolutionary (Cacioppo et al., 2006) theories of loneliness. This finding also has important implications for intervention development, suggesting that loneliness may not be alleviated by the creation of general social connections but that in some cases, it is relationships with specific people or types of people which produce loneliness.

Loneliness can occur due to a multitude of factors and may therefore require different understandings and pathways towards alleviation. Solutions such as befriending groups may be useful for someone who is generally socially satisfied but lonely due to a lack of friends but inappropriate for someone who is lonely due to a spousal bereavement. Schemes such as social prescribing (Reinhardt et al., 2021) may benefit further from considering the specific relationships that give rise to loneliness in some service users. Loneliness is general or pervasive for some individuals, but the relevance of specific relationships and relationship types warrants further study, given that a unidimensional approach continues to dominate loneliness research and practice.

### Age-relevant features of loneliness

This review additionally aimed to shed light on how loneliness is experienced at different life stages. The sources of loneliness may differ across the lifecourse (Child & Lawton, 2019, Qualter et al., 2015) and the use of sensitivity analysis in this work allowed the assessment of how specific age groups impacted the synthesis. Aspects related to the socio-political landscape and personality and identity were not described in child studies, suggesting that these features are less or not relevant to childhood loneliness. Similarly, social comparison, physical and mental health challenges, life transitions, and life experiences were related less by children, possibly because children have had less time to accumulate these experiences. Indeed, research with children who do experience ill health (Wilson et al., 2010) and transitions such as entry into foster care (Mitchell & Kuczynski, 2010) show that they are associated with loneliness. These findings indicate that for children, immediate features of the social network may be more impactful. Meanwhile, for young adults, aspects of relocation appeared to be particularly important. Young adulthood often involves a first move out of the family home and potential subsequent moves to facilitate work or study. Relocation may also carry with it developmentally salient drivers of loneliness such as isolation, increased responsibility, and identity issues (Hopmeyer et al., 2022) which were also implicated in loneliness in this review.

Various aspects of the socio-political landscape were solely attributable to older adults. Bereavement, separation from partner, and reduced opportunities for interaction were also particularly relevant to this age group, in line with a recent review of quantitative longitudinal research (L. Dahlberg et al., 2022). It appears that for children, wider societal factors are less relevant to loneliness while for older adults they are highly relevant, as are experiences related to ageing such as declines in independence and loss of loved ones. For young adults, meanwhile, it may be age-normative transitions such as relocation which are more central. However, the inclusion of only two studies of young adults may have prevented clearer insights into this age group. Additionally, the value and salience of relationship types may vary with age; the synthesis indicated that for older adults, for example, family relationships were particularly relevant, with adult children commonly discussed.

It appears that some features of loneliness are relatively invariant across the lifespan, while others are related to contextual aspects of certain life stages. Interpersonal aspects of loneliness and its aversive, emotional, cognitive, and perceptual aspects appeared to be experienced by lonely people of various ages. However, the relevance of contextual aspects varied. Physical health challenges and bereavement appeared to impact loneliness when they were experienced, but they were more commonly described by older adults. On the other hand, the impact of socio-political context was described less by children. This aligns with recent quantitative research showing that it is the unequal distribution of experiences and resources which explains age differences in loneliness (Hawkley et al., 2022). The current review also found that loneliness could follow from an accumulation of life events, pointing to the relevance of a life-course approach for understanding loneliness. While this review offers a thorough synthesis of research including participants from childhood to old age, studies utilizing longitudinal or cohort approaches would also be valuable to this end. Additionally, most age-specific research in this area appears to focus on older adults, leaving gaps in our knowledge of loneliness in other life stages, particularly middle age. However, the present findings provide a heightened understanding of aspects of loneliness which are more pertinent to specific age groups. This may inform research aiming to understand the dynamics, correlates, and consequences of loneliness at different life stages.

### Limitations and future directions

While the findings of this systematic synthesis further our understanding of the lived experience of loneliness across the lifespan, there are some limitations. Studies of specific clinical or adjacent populations were not included and aspects of loneliness which are distinctive to some specific populations may therefore be underrepresented. This was done to offer a general synthesis which can be further informed by insights from specific populations rather than subsuming and potentially obscuring aspects which may be specific to them; individuals with health difficulties and disabilities were also represented through studies not focusing on specific clinical groups. However, as the findings of this review also represent the varied circumstances, aside from clinical conditions, which impact loneliness, removing this exclusion criterion may be another appropriate approach for future work. This review also included only studies published in English in peer-reviewed journals and may overlook studies in other forums and languages. Additionally, the lack of studies specific to middle-aged adults precluded specific analysis of this age group. Future research might usefully focus on this age group, as well as understudied dynamics of loneliness including perceptual alterations, the relevance of socio-political context, age-related features of lived experiences, and loneliness that relates to specific relationships or relationship types compared to more pervasive forms.

## Conclusion

Loneliness is a prevalent and harmful experience which is receiving a wealth of public, policy, and research attention. Understanding the lived experience of loneliness is central to this work given that it is a fundamentally subjective experience which is common at various stages of life. This systematic review and thematic synthesis illustrates key features of loneliness experiences in people aged from 7 to 103 years old. It appears that loneliness comprises many facets but can be described in terms of three overarching features: loneliness is (1) a psychologically mediated but contextually grounded experience, which (2) centres on feelings of painful disconnection contrasted against a backdrop of meaningful connection, and (3) can be felt in a general, more pervasive sense or can relate to a lack or loss of specific relationships and types. Additionally, it appears that while most features are not specific to a given life stage, some are more pertinent at different stages of the life course, such as functional decline in older adulthood. In providing this detailed synthesis, this study delineates key aspects of loneliness experiences across the lifespan.

## Supplementary Material

Supplemental Material
