# Supplementary material for: Experiences of Loneliness Across the Lifespan: A Systematic Review and Thematic Synthesis of Qualitative Studies
Source: Int J Qual Stud Health Well-being. 2023 Jun 16;18(1):2223868. doi: 10.1080/17482631.2023.2223868 (PMC10281437; doi:10.1080/17482631.2023.2223868)
Supplement: Supplemental Material [file ZQHW_A_2223868_SM4499.zip › Supplementary files/Additional File 1 PRESS Checklist_clean.docx]

# Additional File 1: PRESS Checklist

***PRESS Guideline* — Search Submission & Peer Review Assessment**

**SEARCH SUBMISSION: THIS SECTION TO BE FILLED IN BY THE SEARCHER**

| Searcher: Phoebe McKenna-Plumley | Email: pmckennaplumley01@qub.ac.uk |  |
| --- | --- | --- |
| Date submitted: 29/10/2020 | Date requested by: 05/11/2020 | *[Maximum = 5 working days]* |

**Systematic Review Title:**

Experiences of loneliness: A systematic review and thematic synthesis of qualitative literature

This search strategy is …

| X | My PRIMARY (core) database strategy — First time submitting a strategy for search question and database |
| --- | --- |
|  | My PRIMARY (core) strategy — Follow-up review NOT the first time submitting a strategy for search question and database. If this is a response to peer review, itemize the changes made to the review suggestions |
|  | SECONDARY search strategy— First time submitting a strategy for search question and database |
|  | SECONDARY search strategy — NOT the first time submitting a strategy for search question and database. If  this is a response to peer review, itemize the changes made to the review suggestions |

(Note: Strategy was previously submitted for PsycINFO)

**Database**

(i.e., MEDLINE,CINAHL…): *[mandatory]*

MEDLINE

**Interface**

(i.e., Ovid, EBSCO…): *[mandatory]*

Ovid

**Research Question**

(Describe the purpose of the search) *[mandatory]*

How do people describe their experiences of loneliness?

**PICO Format**

(Outline the PICOs for your question — i.e., Patient, Intervention, Comparison, Outcome, and Study Design — as applicable)

To suit this qualitative evidence synthesis, the SPIDER tool (Cooke, Smith, & Booth, 2012) was used, including the following criteria: Sample, Phenomenon of Interest, Design, Evaluation, Research type

| **S** | Individuals of any age who describe experiences of loneliness, not including specific clinical populations |
| --- | --- |
| **PI** | Loneliness |
| **D** | Any qualitative research design e.g. interview, focus group, etc. Mixed-methods designs which include qualitative methods will be included if the qualitative findings are reported separately. |
| **E** | Descriptions of experiences of loneliness |
| **R** | Primary qualitative research |

**Inclusion Criteria**

(List criteria such as age groups, study designs, etc., to be included) *[optional]*

All age groups will be included. Databases will be searched from date of inception. Studies which focus on qualitatively studying experiences of loneliness will be included.

**Exclusion Criteria**

(List criteria such as study designs, date limits, etc., to be excluded) *[optional]*

The exclusion criteria are as follows:

1. Studies not meeting the inclusion criteria (defined in SPIDER table above)
2. Studies not published in English
3. Quantitative studies with no qualitative component
4. Studies of clinical populations
5. Studies which report solely on objective phenomena such as social isolation rather than the subjectively perceived experience of loneliness
6. Studies in which the primary focus or one of the primary focuses is not experiences of loneliness

Papers will be deemed to focus sufficiently on experiences of loneliness if studying these experiences is a key aspect (such as a central aim/objective) of the work rather than simply a part of the output. The decision to exclude articles which do not primarily or equally focus on these experiences was made in order to capture experiences identified as loneliness by participants as much as possible, rather than related phenomena which may be grouped and labelled retrospectively as loneliness by researchers, and to gather meaningful data about loneliness experiences specifically.

**Was a search filter applied?**

YES X ~~NO~~

**If YES, which one(s) (e.g., Cochrane RCT filter, PubMed Clinical Queries filter)? Provide the source if this is a published filter.** *[mandatory if YES to previous question* — *textbox]*

Filters for studies published in English will be used where available.

Other notes or comments you feel would be useful for the peer reviewer? ***[optional]***

We are specifically interested in experiences of loneliness as described by study participants, rather than other related experiences or experiences which may be labelled as loneliness by researchers. An important distinction here is that loneliness is a subjectively experienced feeling which is separate and can exist separately from objective experiences like being socially isolated. We are interested in synthesising qualitative research which focuses on subjective experiences of loneliness in non-clinical populations.

Following the original PRESS recommendations made on 13/10/2020 and discussion about the search strategy, a number of appropriate and useful subject and free-text terms from the Pearl Harvesting Search Framework synonym ring for qualitative research have been added to capture qualitative research, namely:

Focus Groups/

Personal Narrative/

Grounded Theory/

“discourse analysis”.ti,ab,id.

"case stud*".ti,ab,id.

"audio record*".ti,ab,id.

"audiorecord*".ti,ab,id.

lifeworld*.ti,ab,id.

"life world*".ti,ab,id.

"life-world*".ti,ab,id.

"constant comparative".ti,ab,id.

"constant comparison".ti,ab,id.

"biographical method*".ti,ab,id.

"open-ended".ti,ab,id.

"open ended".ti,ab,id.

As described in meetings, test searches using Patient Isolation/ and Quarantine/ limited to NOT loneliness search terms (in order to exclude studies that would already be captured by the search) did not return unique papers that would be included and primarily returned research on clinical populations. Accordingly, these terms have not been added. Similarly, further test searching using Social Isolation/ and related free-text search terms (e.g. social engagement) limited to NOT loneliness search terms also does not appear to yield unique, eligible papers, given that eligible studies must focus on experiences of loneliness specifically.

The search has been further refined in line with guidance from these meetings, for example searching for free-text search terms in title, abstract, and keyword fields.

Information regarding the above has now been added to the systematic review protocol.

A third set of search terms related to “outcomes” (e.g. AND experiences OR perceptions) was not included given that experiences of loneliness could be described simply as “loneliness” (e.g. “Participants were interviewed about loneliness”) and therefore this addition may be too restrictive.

Please copy and paste your search strategy here, exactly as run, including the number of hits per line. ***[mandatory]***

**(Add more space, as necessary.)**

Databases to be searched:

MEDLINE, PsycINFO, SCOPUS, Child Development & Adolescent Studies, ERIC, Sociological Abstracts, IBSS, CINAHL

Search strategy as run in MEDLINE ALL (OVID) on 28/10/2020:

| **#** | **Searches** | **Results** |
| --- | --- | --- |
| 1 | Loneliness/ | 3860 |
| 2 | lonel*.ti,ab,id. | 7591 |
| 3 | ("social* isolat*") ADJ2 (perce*).ti,ab,id. | 140 |
| 4 | **combine 1, 2, 3 with OR** | 8600 |
| 5 | Qualitative Research/ | 57585 |
| 6 | qualitative*.ti,ab,id. | 278996 |
| 7 | "mixed method*".ti,ab,id. | 23042 |
| 8 | "mixed-method*".ti,ab,id. | 23042 |
| 9 | Interview/ | 29358 |
| 10 | interview*.ti,ab,id. | 364484 |
| 11 | Focus Groups/ | 30604 |
| 12 | "focus group*".ti,ab,id. | 47974 |
| 13 | "thematic analysis".ti,ab,id. | 20479 |
| 14 | Personal Narrative/ | 5185 |
| 15 | "narrative analysis".ti,ab,id. | 1196 |
| 16 | "narrative approach".ti,ab,id. | 788 |
| 17 | Grounded Theory/ | 1764 |
| 18 | "grounded theory".ti,ab,id. | 11769 |
| 19 | "phenomenological analysis".ti,ab,id. | 2430 |
| 20 | "phenomenological approach".ti,ab,id. | 2437 |
| 21 | ethnograph*.ti,ab,id. | 10991 |
| 22 | "discourse analysis".ti,ab,id. | 1754 |
| 23 | "content analysis".ti,ab,id. | 29083 |
| 24 | "lived experience".ti,ab,id. | 3848 |
| 25 | "group discussion".ti,ab,id. | 3293 |
| 26 | "case stud*".ti,ab,id. | 98207 |
| 27 | "audio record*".ti,ab,id. | 6731 |
| 28 | "audiorecord*".ti,ab,id. | 368 |
| 29 | lifeworld*.ti,ab,id. | 481 |
| 30 | "life world*".ti,ab,id. | 476 |
| 31 | "life-world*".ti,ab,id. | 476 |
| 32 | "constant comparative".ti,ab,id. | 3193 |
| 33 | "constant comparison".ti,ab,id. | 1573 |
| 34 | "biographical method*".ti,ab,id. | 27 |
| 35 | "open-ended".ti,ab,id. | 14653 |
| 36 | "open ended".ti,ab,id. | 14653 |
| 37 | **combine 5, 6, 7, 8, 9, 10, 11, 12, 13, 14, 15, 16, 17, 18, 19, 20, 21, 22, 23, 24, 25, 26, 27, 28, 29, 30, 31, 32, 33, 34, 35, 36 with OR** | 725063 |
| 38 | **4 AND 37** | 1923 |
| 39 | **Limit 38 to English** | 1800 |

**PEER REVIEW ASSESSMENT: THIS SECTION TO BE FILLED IN BY THE REVIEWER**

|  | Reviewer: Dr Ciara Keenan | Email:Ciara.keenan@nuigalway.ie | Date completed:28/10/2020 | | |
| --- | --- | --- | --- | --- | --- |
|  |  |  |  | | |
|  | **1. TRANSLATION** |  |  | | |
| A -­‐No revisions | | ☐ |  |  |  |
| B -­‐ Revision(s) suggested | | ☐ |  |  |  |
| C -­‐ Revision(s) required | | ☐ |  |  |  |

If “B” or “C,” please provide an explanation or example:

**2. BOOLEAN AND PROXIMITY OPERATORS**

| A -­‐No revisions | ☐ |
| --- | --- |
| B -­‐ Revision(s) suggested | ☐ |
| C -­‐ Revision(s) required | ☐ |

If “B” or “C,” please provide an explanation or example:

**3. SUBJECT HEADINGS**

| A -­‐No revisions | ☐ |
| --- | --- |
| B -­‐ Revision(s) suggested | ☐ |
| C -­‐ Revision(s) required | ☐ |

If “B” or “C,” please provide an explanation or example:

**4. TEXT WORD SEARCHING**

| A -­‐No revisions | ☐ |
| --- | --- |
| B -­‐ Revision(s)suggested | ☐ |
| C -­‐ Revision(s) required | ☐ |

If “B” or “C,” please provide an explanation or example:

**5. SPELLING, SYNTAX, AND LINE NUMBERS**

| A -­‐No revisions | ☐ |
| --- | --- |
| B -­‐ Revision(s)suggested | ☐ |
| C -­‐ Revision(s) required | ☐ |

If “B” or “C,” please provide an explanation or example:

**6. LIMITS AND FILTERS**

| A -­‐No revisions | ☐ |
| --- | --- |
| B -­‐ Revision(s) suggested | ☐ |
| C -­‐ Revision(s) required | ☐ |

If “B” or “C,” please provide an explanation or example:

OVERALL EVALUATION (Note: If one or more “revision required” is noted above, the response below must be “revisions required”.)

| A -­‐No revisions | ☐ |
| --- | --- |
| B -­‐ Revision(s) suggested | ☐ |
| C -­‐ Revision(s) required | ☐ |

Additional comments:
