# Supplementary material for: Experiences of Loneliness Across the Lifespan: A Systematic Review and Thematic Synthesis of Qualitative Studies
Source: Int J Qual Stud Health Well-being. 2023 Jun 16;18(1):2223868. doi: 10.1080/17482631.2023.2223868 (PMC10281437; doi:10.1080/17482631.2023.2223868)
Supplement: Supplemental Material [file ZQHW_A_2223868_SM4499.zip › Supplementary files/Additional File 2 MEDLINE Search Strategy.docx]

**Additional File 2: MEDLINE Search Strategy**

Search strategy for MEDLINE ALL (OVID)

| **#** | **Searches** |
| --- | --- |
| 1 | exp Loneliness/ |
| 2 | lonel*.ti,ab,kw. |
| 3 | ("social* isolat*") ADJ6 (perce*).ti,ab,kw. |
| 4 | **combine 1, 2, 3 with OR** |
| 5 | exp Qualitative Research/ |
| 6 | qualitative*.ti,ab,kw. |
| 7 | "mixed method*".ti,ab,kw. |
| 8 | "mixed-method*".ti,ab,kw. |
| 9 | exp Interview/ |
| 10 | interview*.ti,ab,kw. |
| 11 | exp Focus Groups/ |
| 12 | "focus group*".ti,ab,kw. |
| 13 | "thematic analysis".ti,ab,kw. |
| 14 | exp Personal Narrative/ |
| 15 | "narrative analysis".ti,ab,kw. |
| 16 | "narrative approach".ti,ab,kw. |
| 17 | exp Grounded Theory/ |
| 18 | "grounded theory".ti,ab,kw. |
| 19 | "phenomenological analysis".ti,ab,kw. |
| 20 | "phenomenological approach".ti,ab,kw. |
| 21 | ethnograph*.ti,ab,kw. |
| 22 | "discourse analysis".ti,ab,kw. |
| 23 | "content analysis".ti,ab,kw. |
| 24 | "lived experience".ti,ab,kw. |
| 25 | "group discussion".ti,ab,kw. |
| 26 | "case stud*".ti,ab,kw. |
| 27 | "audio record*".ti,ab,kw. |
| 28 | "audiorecord*".ti,ab,kw. |
| 29 | lifeworld*.ti,ab,kw. |
| 30 | "life world*".ti,ab,kw. |
| 31 | "life-world*".ti,ab,kw. |
| 32 | "constant comparative".ti,ab,kw. |
| 33 | "constant comparison".ti,ab,kw. |
| 34 | "biographical method*".ti,ab,kw. |
| 35 | "open-ended".ti,ab,kw. |
| 36 | "open ended".ti,ab,kw. |
| 37 | **combine 5, 6, 7, 8, 9, 10, 11, 12, 13, 14, 15, 16, 17, 18, 19, 20, 21, 22, 23, 24, 25, 26, 27, 28, 29, 30, 31, 32, 33, 34, 35, 36 with OR** |
| 38 | **4 AND 37** |
